# Condensed Tannins in White Clover (*Trifolium repens*) Foliar Tissues Expressing the Transcription Factor TaMYB14-1 Bind to Forage Protein and Reduce Ammonia and Methane Emissions *in vitro*

**DOI:** 10.3389/fpls.2021.777354

**Published:** 2022-01-06

**Authors:** Marissa B. Roldan, Greig Cousins, Stefan Muetzel, Wayne E. Zeller, Karl Fraser, Juha-Pekka Salminen, Alexia Blanc, Rupinder Kaur, Kim Richardson, Dorothy Maher, Zulfi Jahufer, Derek R. Woodfield, John R. Caradus, Christine R. Voisey

**Affiliations:** ^1^Grasslands Research Centre, AgResearch Ltd, Palmerston North, New Zealand; ^2^PGG Wrightson Seeds Ltd, Palmerston North, New Zealand; ^3^ARS-USDA, US Dairy Forage Research Center, Madison, WI, United States; ^4^Riddet Institute, Massey University, Palmerston North, New Zealand; ^5^Natural Chemistry Research Group, University of Turku, Turku, Finland; ^6^AgroParis Tech, Paris, France; ^7^Grasslanz Technology Ltd, Palmerston North, New Zealand

**Keywords:** proanthocyanidin, prodelphinidin, *Trifolium repens*, protein protection, climate change, ammonia, plant breeding, pasture bloat

## Abstract

Grazing ruminants contribute to global climate change through enteric methane and nitrous oxide emissions. However, animal consumption of the plant polyphenolics, proanthocyanidins, or condensed tannins (CTs) can decrease both methane emissions and urine nitrogen levels, leading to reduced nitrous oxide emissions, and concomitantly increase animal health and production. CTs are largely absent in the foliage of important temperate pasture legumes, such as white clover (*Trifolium repens*), but found in flowers and seed coats. Attempts at enhancing levels of CT expression in white clover leaves by mutagenesis and breeding have not been successful. However, the transformation of white clover with the TaMYB14-1 transcription factor from *Trifolium arvense* has resulted in the production of CTs in leaves up to 1.2% of dry matter (DM). In this study, two generations of breeding elevated foliar CTs to >2% of DM. The CTs consisted predominantly of prodelphinidins (PD, 75–93%) and procyanidins (PC, 17–25%) and had a mean degree of polymerization (mDP) of approximately 10 flavan-3-ol subunits. *In vitro* studies showed that foliar CTs were bound to bovine serum albumin and white clover proteins at pH 6.5 and were released at pH 2.-2.5. Using rumen *in vitro* assays, white clover leaves containing soluble CTs of 1.6–2.4% of DM significantly reduced methane production by 19% (*p* ≤0.01) and ammonia production by 60% (*p* ≤ 0.01) relative to non-transformed wild type (WT) controls after 6 h of incubation. These results provide valuable information for further studies using CT expressing white clover leaves for bloat prevention and reduced greenhouse gas emissions *in vivo*.

## Introduction

Anthropogenic greenhouse gas emissions pose a serious environmental challenge due to their effects on climate change, with methane 30 times more effective than carbon dioxide in trapping heat in the atmosphere (Stocker et al., 2013), and nitrous oxide (N_2_O) emissions being a key player in ozone depletion (Revell et al., 2015). The livestock supply chain produces approximately 14.5% of global gas emissions, 44% of which is methane, with the beef and dairy cattle sectors being the major contributors (Gerber et al., 2013). Much of the N_2_O emissions from livestock production systems originate from urine patches and animal excreta (López-Aizpún et al., 2020). Ruminant livestock fed on lush pastures rich in soluble proteins can also suffer from bloat. This potentially fatal digestive disorder (Wang et al., 2012) is caused by gasses released during forage fermentation that can become trapped in a stable foam in the rumen, preventing eructation and affecting lung and heart function.

**FIGURE 1:**
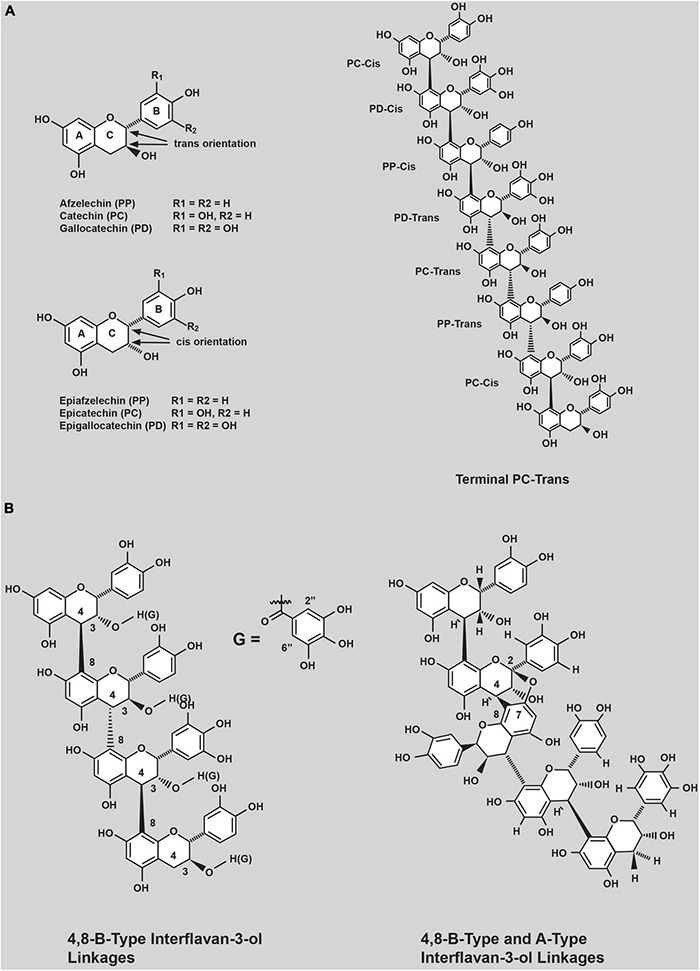
**(A)** Common flavan-3-ol subunits contained in condensed tannins occurring in forage plants and **(B)** examples of connectivity of common flavan-3-ol subunits observed in purified condensed tannins (CTs) from white clover primary (T_0_) transgenic plants.

Condensed tannins (CTs, proanthocyanidins) are polyphenolic compounds (Dixon and Sarnala, 2020) that occur abundantly in many vascular plants and have proven efficacy in reducing ruminant methane emissions (Tan et al., 2011; Huyen et al., 2016; Ku-Vera et al., 2020) and preventing pasture bloat (McMahon et al., 2000; Mueller-Harvey et al., 2019; Kelln et al., 2021). CTs are formed through the covalent linkage of flavan-3-ol subunits in their *trans* (afzelechin, catechin, and gallocatechin) or *cis* (epiafzelechin, epicatechin, and epigallocatechin) configurations (Figure 1A). Afzelechin and epiafzelechin are referred to as propelargonidin subunits, catechin and epicatechin as procyanidin (PC) subunits, and gallocatechin and epigallocatechin as prodelphinidin (PD) subunits. Representative structures of these subunits are shown in Figure 1A. The flavan-3-ol subunits of CTs can assemble *via* different bonds. The most common connectivity reported is the 4,8-B-type linkage (Figure 1B, left panel), involving a single covalent linkage between C-4 and C-8 of adjacent flavan-3-ol subunits (Zeller, 2019). The second class of interflavan-3-ol subunit linkage in CTs comprises two covalent linkages between adjacent flavan-3-ol subunits and is referred to as the A-type linkage. In this interflavan-3-ol bond arrangement, covalent bonds are formed between C8 and the oxygen atom connected to C7 of the A-ring of one flavan-3-ol subunit to the C4 and C2 atoms of the C-ring, respectively, of the adjacent flavan-3-ol subunit (Figure 1B, right panel).

Protein protection and release are key biological activities of CTs in ruminants (Min et al., 2003) where both CT polymer size and pH are primary factors that affect CT-protein complexation and release (Saminathan et al., 2014). Protein complexation by CTs is affected by both the CT subunit composition and their mean degree of polymerization (mDP) (Ropiak et al., 2017). Generally, in plants containing PCs, PDs and their mixtures, protein precipitation is stronger with higher PD content and with higher mDP (Leppä et al., 2020). However, there are also species-specific, and thus CT-specific, differences in protein precipitation, as plants produce highly variable mixtures of oligomeric and polymeric CTs with thousands of individual structures (Leppä et al., 2018, 2020).

The binding of condensed tannins to proteins in the rumen prevents their metabolism to ammonia and fatty acids. Reduced proteolysis in the rumen also decreases urinary nitrogen loss (Waghorn, 2008; Dschaak et al., 2011) and prevents the foam formation that causes bloat (McMahon et al., 2000). The pH reduction that occurs as a digesta move from the rumen (pH 6-7) to the abomasum (pH 2-3) causes dissociation of the CT-protein complex (Dentinho and Bessa, 2016), and the proteins are converted to peptides and amino acids available for absorption in the lower gastrointestinal tract (Naumann et al., 2017), resulting in greater feed utilization and increased animal productivity (meat, milk, and wool) (Waghorn and McNabb, 2003). These collective benefits highlight the importance of developing forages that contain appropriate amounts and types of CTs in their leaves.

Studies on livestock benefits from feeding CT-containing forages have focused on temperate legumes, such as *Lotus pedunculatus*, *Hedysarum coronarium*, and *Onobrychis viciifolia* (Waghorn and Shelton, 1995; Waghorn et al., 1998; Molan et al., 2001) that produce CTs, constituting up to 8% of DM. Despite this advantage, the agronomic utility of these species is marred by poor persistence under year-round grazing and low dry matter yield (Waghorn et al., 1998; Widdup et al., 2004). By contrast, species which perform well in grazing systems, such as perennial ryegrass (*Lolium perenne*) and white clover (*Trifolium repens*), produce little or no CTs in leaves (Roldan et al., 2020; Kagan, 2021). Nevertheless, white clover does produce CTs in the trichomes on the abaxial epidermis of leaves, and relatively high prodelphinidin concentrations in mature inflorescences (>3% of DM), indicating the potential to exploit this valuable trait. Plant breeding to increase flowering can increase CTs, but concentrations were too low to improve animal performance (Woodfield et al., 1998; Burggraaf et al., 2006). However, a breakthrough in the understanding of the regulation of CT expression in *Trifolium arvense*, which produces CTs in its leaves, revealed that an R2R3 MYB transcription factor (TF; *Ta-MYB14-1*) was essential for regulating CT leaf expression in that species. Three white clover homologs (*Tr-MYB14-1, 2*, and 3) with >95% amino acid identity with *Ta-MYB14* were not expressed in cDNA libraries of wild type white clover leaves (CT negative) or petals (CT positive), indicating no correlation with CT synthesis (Hancock et al., 2012). However, expression of *Ta-MYB14-1* in white clover and alfalfa (*Medicago sativa*) was sufficient to induce CT accumulation in foliar tissues to up to 1.8% of dry matter (DM) (Hancock et al., 2012), offering a unique opportunity to develop persistent forage legumes that could reduce methane emissions and prevent bloat in ruminants.

Test crosses by Roldan et al. (2020), using plants developed by Hancock et al. (2012) have shown that significant gains (3.6-fold on average) in CT accumulation were possible when parental lines were crossed with plants containing the endogenous white clover R2R3 MYB TF, *Tr-RED LEAF* (Roldan et al., 2020). This R2R3 MYB (at the so-called “R” locus) is known to regulate the expression of white clover leaf markings, such as “red leaf,” “red midrib,” and “red fleck” caused by an epidermal accumulation of the polyphenol anthocyanin, which is also derived from the phenolpropanoid pathway (Dixon et al., 2013; Albert et al., 2015).

The expression of *TaMYB14-1* in white clover germplasm containing the *Tr-RED LEAF* “R” locus should enable the accumulation of CTs in leaves at the required concentration (>1.5% of DM) for demonstrable effects on protein binding and methane emissions in rumen fluid *in vitro*. The transformation of *TaMYB14-1* into a modern large-leafed commercial white clover variety (cv. Mainstay), containing the *Tr-RED LEAF* “R” locus, is described, followed by the crossing of individual primary transformants into genetically diverse elite cv. Mainstay genotypes toward the development of a robust white clover variety with efficacious levels of CT in leaves. The aim here is to quantify CT concentration and composition in white clover leaves, and the impacts of breeding and allele frequency on CT levels in different generations of plants. Finally, we tested the hypotheses that white clover leaf CTs are able to bind and release protein at the physiological pH of the rumen and intestine of ruminants, respectively, and are capable of reducing methane in rumen fluid in an *in vitro* gas production method.

## Materials and Methods

### Transformation of White Clover, Plant Crossing, and Segregation Analysis

All plant transformation and subsequent crossing experiments used the seed of the commercial white clover cultivar “Grasslands Mainstay” (accession No. C27068), obtained from the Margot Forde Germplasm Centre, AgResearch Ltd, Palmerston North, New Zealand. This large-leaved cultivar contains the “red fleck” anthocyanin mark on the adaxial epidermal leaf surface (Supplementary Figure 1B). The primary transgenic events were produced by *Agrobacterium*-mediated transformation using the binary vector pART27-TaMYB14-1. *TaMYB14-1* was PCR amplified from genomic DNA of *T. arvense* and is an allelic variant of NCBI accession JN049641.1 as previously described (Roldan et al., 2020). *TaMYB14-1* was fused 5′ to the Cauliflower Mosaic Virus (CaMV) 35S promoter and 3′ to the octopine synthase terminator in the T-DNA of plant binary vector pART27 (Supplementary Figure 1A). It was co-selected with the neomycin phosphotransferase gene (*NptII*) bordered 5′ by the nopaline synthase (NOS) promoter and 3′ by the NOS terminator. Over 25,000 white clover cotyledonary explants were inoculated with *A. tumefaciens* GV3101 as described in Supplementary Methods section “White Clover Transformation.” Transgenic plants were initially screened for *TaMYB14-1* by PCR, and CT-expressing plants were identified by staining leaflets using the chromogenic reagent dimethylaminocinnamaldehyde (Li et al., 1996) (DMACA; Supplementary Methods section “White Clover Transformation”). *TaMYB14-1* copy number in primary transformants was determined by Southern blot hybridization and droplet digital PCR (Supplementary Methods section “Determination of *TaMYB14-1* Copy Number”). *TaMYB14-1* zygosity was also determined by ddPCR. Transgenic plants were transferred to pots containing soil and maintained under glasshouse conditions as described in Supplementary Methods section “Plant Maintenance.” Protocols for seed production and segregation of *TaMYB14-1* in progeny seed are given in Supplementary Methods section “Breeding Strategy and Segregation Analysis.”

### Quantification of Soluble and Insoluble Condensed Tannins in White Clover Leaves

Trifoliate leaf samples (petioles excluded) were taken from plants growing in potted soil under glasshouse conditions. The temperature ranged between 15 and 22*^o^*C, and relative humidity was between 75 and 85%. Samples were taken from mature primary transgenic plants and from progeny plants 8 and 12 weeks after sowing, and then freeze-dried and milled to a fine powder using a bead mill homogenizer (OMNI Bead Ruptor, VWR OMNI International Inc., CA). Two technical replicates were assayed for each sample. Methods to extract and quantify soluble CTs were as previously described (Roldan et al., 2020) with modifications as detailed in Supplementary Methods section “Quantification of Soluble and Insoluble CTs in White Clover Leaves.”

### Analysis of Condensed Tannins by HSQC NMR Spectroscopy

The preparation of leaf samples for chemical composition analysis, and the extraction and purification of CTs from CTB-T_0_, CTF-T_0_, and CTG-T_0_ are described in Supplementary Methods section “Preparation of Leaf Samples for CT Composition Analysis” and section “Extraction and Purification of CTs From T_0_ Transgenic Plants and Their Progeny,” respectively. Samples from five independent clones per T_0_ event were repeatedly harvested and freeze dried to obtain approximately 20 g of bulked material from each T_0_ event. CT composition of the T_0_ events was analyzed using ^1^H, ^13^C, and ^1^H-^13^C HSQC NMR spectroscopy (Zeller et al., 2015a). The ^1^H, ^13^C, and ^1^H-^13^C HSQC NMR spectra for the CT fractions used in the precipitation studies were recorded at 27°C on a Bruker BioSpin DMX-500 (^1^H 500.13 MHz, ^13^C 125.76 MHz) instrument equipped with TopSpin 3.5 software and a cryogenically cooled 5-mm TXI 1H/13C/15N gradient probe in inverse geometry. Spectra were recorded in DMSO-*d*_6_ and were referenced to the residual signals of DMSO-*d*_6_ (2.49 ppm for ^1^H and 39.5 ppm for ^13^C spectra). Spectra were obtained using the standard Bruker pulse program “hsqcegtpsi” using the acquisition and processing parameters previously described (Zeller et al., 2015a).

### Analysis of Condensed Tannin Composition Using Waters Xevo UPLC-DAD-MS/MS System

The CT composition was also analyzed using the Waters Xevo UPLC-DAD-MS/MS system (Salminen, 2018). The T_0_ samples were from clones of plants described for HSQC NMR spectroscopy above, while samples from the BC1, BC2, and T2 progenies were from plants described for quantification of CTs. Here, the composition of CTs in samples was measured with group-specific PC and PD analytics, specifically developed for Waters Xevo UPLC-DAD-MS/MS system (Engström et al., 2014; Salminen, 2018). This method separately quantifies PC and PD units present in complex mixtures of PC/PD oligomers and polymers. It detects both terminal and extension units of PCs and PDs separately as well, thus enabling the calculation of the mean degree of polymerization for the CTs present in the analyzed sample. Extracts were analyzed as described in Malisch et al. (2015) and James et al. (2017).

### Binding and Dissociation of Proteins by White Clover Leaf Condensed Tannins

Bovine serum albumin (BSA) and white clover proteins were used in the *in vitro* CT-protein binding and a dissociation assay with CTs, extracted and purified as described in Supplementary Methods section “Preparation of Leaf Samples for CT Composition Analysis,” from each primary transgenic event (CTB-T_0_, CTF-T_0_, and CTG-T_0_). The BSA protein stock was prepared by dissolving the BSA in 50 mM 2-(N-morpholino) ethanesulfonic acid (MES), pH 6.5, to a concentration of 10 mg/ml. Similarly, the stock solution (10 mg/ml) of purified CT (described in Supplementary Methods section “Extraction and Purification of CTs From T_0_ Transgenic Plants and Their Progeny”) was prepared by dissolving lyophilized CTs in a 50-mM MES buffer, pH 6.5. For the assay, 20 μl of BSA stock was mixed with 20 μl of purified CT stock and added with a 50-mM MES buffer, pH 6.5, to a final volume of 200 μl. Crude protein from non-transgenic white clover leaves (Mainstay, genotype HS227/3 R2) was obtained following published procedures (Zeller et al., 2015b) and described in Supplementary Methods section “Extraction and Quantification of White Clover Protein.” The volume used in the assay was adjusted based on the concentration of the protein extract to ensure that, after mixing 20 μl of the CT stock, the final concentration of protein and CT in a 200-μl assay was 1 mg/ml in 50 mM MES, pH 6.5. For the protein-binding assay with crude CT extract, 40 μl of 10-mg/ml crude CT stock [described in Supplementary Methods section “Extraction and Quantification of Soluble and Insoluble CTs in White Clover Leaves”] was used to produce a final concentration of 2 mg/ml CTs and 1 mg/ml protein.

The protein/CT mixtures were incubated on ice for 20 min, and, after centrifugation at 15,000 x *g* for 10 min, the supernatant was transferred to a fresh tube. The surface of the pellet and the walls of the incubation tubes were rinsed gently with a 50-mM MES buffer, pH 6.5, without disturbing the pellet to remove any residual protein still in solution. The precipitate was resuspended in a 200-μl MES buffer (pH 2. or 2.5), mixed by a gentle vortex, and incubated as above. After centrifugation, the supernatant was transferred to a fresh tube, and the precipitate was again resuspended in a 50-mM MES buffer, pH 2.0 or 2.5. The protein concentration in the different fractions was quantified using a Qubit^®^ Fluorometer. A 25-μl aliquot from the supernatant and the pellet was then prepared for sodium dodecyl sulfate-polyacrylamide gel electrophoresis (SDS-PAGE) by adding an equal volume of 2x SDS loading dye (Bio-Rad) and heating at 95*^o^*C for 5 min. After the separation in a 1x Tris/Glycine SDS buffer (Bio-Rad) at 150 V for 1 h with a dual color Precision Plus Protein Standard (Bio-Rad), the protein gel was stained with Bio-Safe™ G-50 Stain (Bio-Rad) according to the manufacturer’s instructions for protein visualization. The experiment was conducted two times for each biological replicate.

### *In vitro* Fermentation in Rumen Fluid and End Product Analysis

The substrates tested included WT white clover leaves, WT white clover inflorescences, and leaves harvested from the vegetative clones of two independent genotypes from second-generation progeny (T_2_), homozygous for the transgene (CTG-T_2_, 3755, and 3764). Green leaves of all ages were harvested periodically until a sufficient amount required for the experiments was obtained. Tissue samples were freeze-dried immediately after each harvest, milled, and stored at -20*^o^*C until required. Milled powders from clones of the same genotypes were pooled and CTs quantified as described above. To control for potential effects from a lack of uniformity in the nutritional composition of the substrates, such as acid detergent fiber (ADF), ash, crude protein (CP), lipid, ME neutral detergent fiber (NDF), organic matter digestibility (OMD), and soluble sugars and starch (SSS), materials used in the *in vitro* fermentation assay were analyzed using near-infrared reflectance spectroscopy (NIRS) (Analytical Research Laboratories, Napier, New Zealand).

The milled samples (500 mg) were incubated in rumen fluid (diluted as described below) with and without 50 mg of polyethylene glycol (PEG) 6000 in an automated rumen batch culture system as described (Muetzel et al., 2014). PEG 6000 binds CTs and was used to control for matrix effects. Rumen fluid from two fistulated pasture-fed cows was collected into a pre-warmed insulated flask on the morning of the incubation and prior to the morning feed allocation to increase the uniformity between rumen samples. The rumen fluid was transported to the laboratory within 15 min of collection. The rumen fluid from each donor animal was filtered through a layer of cheese cloth, mixed in equal proportions, and diluted to 25% (v/v) in reduced and a pre-warmed (39°C) bicarbonate buffer (Mould et al., 2005). The medium was then dispensed in 50-ml aliquots into the pre-warmed serum bottles under a constant stream of CO_2_ (Muetzel et al., 2014). The bottles were randomly assigned to the array and placed on a horizontal shaker in a 39°C incubator and connected to the gas analysis system. Total gas and methane production was measured automatically using a pressure sensor and a valve which diverted the produced gasses into a gas chromatograph for quantitation, and to depressurize the bottle. Each fermentation experiment contained 16 bottles (4 substrates, 2 replicates each, 2 bottles/rep (one +PEG and one −PEG) and was repeated three times with rumen fluid from two different donor animals each time (six cows in total).

Independent *in vitro* fermentation assays were conducted to analyze protein degradation products, including ammonia, isovalerate, isobutyrate, acetate, priopionate, and butyrate. The substrates, experimental replication, and incubation conditions were identical to those described for the gas analysis above. At 6 h and 24 h, 1.8 ml of rumen fluid was withdrawn from the bottles with a 16-G needle for ammonia and short chain fatty acid analyses. Samples were centrifuged (21,000 x *g*, 10 min, 4°C) and 900 μl of the supernatant was taken into 100 μl of internal standard solution [19-mM ethyl butyrate in 20% (v/v) phosphoric acid]. Samples were kept at −20°C overnight, thawed, and centrifuged as above. An 800 μl aliquot of the supernatant was transferred into a 2-ml crimp cap gas chromatography vial for analysis of SCFA (Richardson et al., 1989; Attwood et al., 1998). A further 100 μl of the supernatant was transferred into a 96 well plate for the determination of ammonia concentration using the phenol hypochlorite method (Weatherburn, 1967). The experiments were conducted under the conditions stipulated by the AgResearch Grasslands Animal Ethics Committee Approval No. 13398.

### Statistical Analysis

Minitab Statistical Software was used to analyze data including normality of distribution and chi-square goodness of fit test. To test whether the progeny segregated as expected, a chi-square test was used at a 95% confidence level. The effects of CT (expressed as percent reduction in fermentation products) were first determined relative to treatment with PEG 6000 and were tested using one-way ANOVA to determine significant differences among means. Mean comparisons were completed using Tukey’s Multiple Comparison Test (MCT) at *p* ≤ 0.01.

## Results

### TaMYB14-1 White Clover and Condensed Tannin Expression in Leaves

The *TaMYB14-1* gene, expressed from the cauliflower mosaic virus 35S promoter, was stably integrated into the genome of the large-leaved white clover cultivar “Grasslands Mainstay” using *Agrobacterium*-mediated transformation (Supplementary Figures 1A–D). From the 25,000 cotyledons inoculated, 162 plants (0.65% of cotyledons) survived kanamycin selection, and 57 (0.23% of cotyledons) were identified as T-DNA positive by PCR (Supplementary Table 1A). The primary transgenic (T_0_) lines (referred to as transformation events hereafter) were next scored for CT synthesis in a leaflet from a young trifoliate leaf using the chromogenic reagent dimethylaminocinnamaldehyde (DMACA). CT staining was ranked according to an arbitrary score of 1–7 (Supplementary Figure 2), and the 22 strongest (DMACA score ≥ 4) CT-expressing lines (CTA to CTV) were selected for quantitation of soluble CTs in the leaves. Soluble CTs in leaves ranged from 0.22 to 1.53% of DM epigallocatechin equivalents (Supplementary Figure 1E). Southern blot hybridization and droplet digital PCR analyses indicated that 15 of the 22 plants had a single *TaMYB14-1* gene insertion, while the other events had between two and four copies (Supplementary Figure 1E, bottom panel; Supplementary Figure 3B).

### Introgression of Condensed Tannin-Expressing Transformation Events Into Elite Germplasm

White clover is predominantly an outcrossing species, and highly diverse genetically. Repeated crossing between individually transformed events (single genotypes) and genetically diverse, high performing untransformed parents are required to build genetic diversity into the variety, both to prevent inbreeding depression and to select for the progeny with desirable CT levels and composition. The eight highest CT-expressing events were backcrossed to elite non-transformed “Grasslands Mainstay” genotypes to determine if the T-DNA insertion segregated in the BC_1_ progeny in the expected 1:1 (T-DNA +ve: T-DNA −ve) ratio (Supplementary Table 1B). The “Mainstay” parents used in these crosses had been identified as having the highest general combining ability based on 3 years of progeny testing. Seeds were germinated in Petri dishes before planting individually into pots, and CT synthesis in the middle leaflet of the first trifoliate leaf to emerge (4 weeks after planting) was estimated after staining with DMACA. Stain intensity/distribution was scored from 0 to 7 (lowest to highest, Supplementary Figure 2) and a DMACA score of ≥ 1 was used as a proxy for the inheritance of *TaMYB14-1* as previously described (Roldan et al., 2020). Genotypes with imperfect T-DNA segregation ratios, or which produced few flowers were discarded, and the remaining 3 events (CTB-T_0_, CTF-T_0_, and CTG-T_0_; Supplementary Table 1B) were selected for further breeding. The genomic DNA of these events was checked by PCR for the presence of contaminating vector sequences using 12 primer pairs (Supplementary Table 2) that spanned the entire Ti-plasmid used in the transformations (Supplementary Figure 4A). PCR products were only detected from primers targeting the region within the T-DNA borders, suggesting that contaminating vector sequences were not present (Supplementary Figure 4B).

**FIGURE 2:**
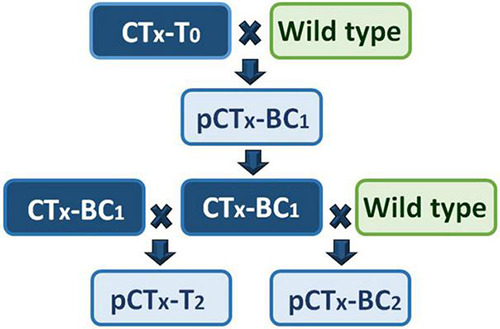
A crossing scheme to generate the first and second generations of progeny. CTx refers to the 3 transgenic events (CTB, CTF, and CTG) used in crossing, T_0_ is a primary transgenic event, BC_1_ is a progeny of a backcross (BC) between primary transgenic and a wild-type (non-transformed) Mainstay white clover, BC_2_ is the progeny of a backcross between BC_1_ and wild-type clover, and T_2_ is the progeny of a cross between two BC_1_ individuals. pCTx refers to the progeny of a specified cross.

**FIGURE 3:**
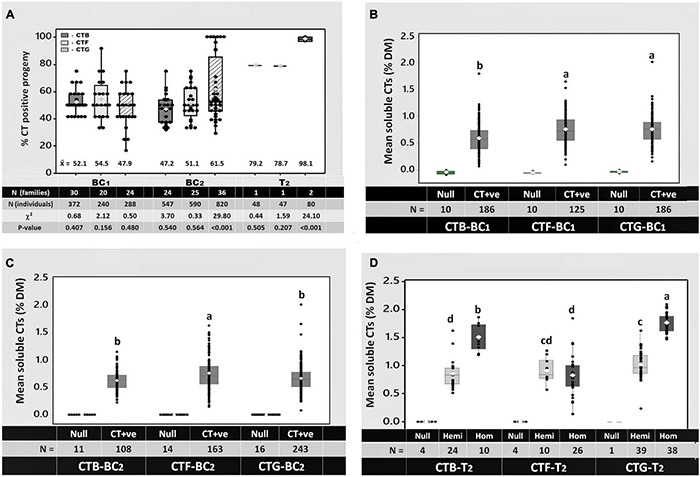
**(A)** Segregation of the condensed tannin leaf trait in the first (BC_1_) and second (BC_2_ and T_2_) generations of CTB, CTF, and CTG progenies, and **(B–D)** quantitation of soluble condensed tannins in the leaves of **(B)** backcross 1 (BC_1_), **(C)** backcross 2 (BC_2_), and **(D)** T_2_ (BC_1_ x BC_1_) progenies. In panel **(A)**, the mean of each group is written below each bar. The number of families and individuals analyzed, as well as chi-square statistics for each group of progenies and corresponding *P*-values, is also provided at the bottom of panel **(A)**. In panels **(B–D)**, each data point shows the mean soluble CTs in the leaves of an individual plant at 8 and 12 weeks after planting, expressed in percent dry matter (% DM, epigallocatechin equivalents). The white diamonds in panels **(B–D)** show the group mean CTs as a percent of DM; different letters on top of bars indicate significant mean differences in each group by Tukey’s Multiple Comparison Test (MCT) *P* ≤ 0.01. The total number (N) of individuals analyzed per group is provided below each bar. In panel **(D)**, the null, hemi (hemizygotes), and the hom (homozygotes) are individuals that contain 0, 1, and 2 copies of the *TaMYB14-1* gene, respectively.

Full-sibling BC_1_ families from reciprocal backcrosses of CTB-T_0_, CTF-T_0_, and CTG-T_0_ to at least 10 non-transformed cv. “Grasslands Mainstay” genotypes were then generated (Figure 2). Seeds (12 per family from each reciprocal cross) were planted, and a leaflet from each seedling was stained with DMACA and scored for CT synthesis as described above. DMACA scores of ≥ 1 were obtained in 52.1, 54.5, and 47.9% of BC_1_ progeny from CTB-T_0_, CTF-T_0_, and CTG-T_0_, respectively (Figure 3A). A chi-square goodness-of-fit test indicated no significant differences between the observed and expected frequencies in BC_1_ (*p* ≥ 0.16; Figure 3A). A total of 6–7 *TaMYB14-1*-positive BC_1_ genotypes from each of the 3 transformation events, selected for both high CT levels and growth performance, were backcrossed to at least 16 additional elite wild-type cv. “Grasslands Mainstay” genotypes to generate a minimum of 33 backcross (BC_2_) full sibling families per event. The objective of the second backcross, which used different “Grasslands Mainstay” genotypes, was to increase genetic diversity in the progeny and produce an agronomically superior population with high CT expression (≥1%) for future plant breeding. DMACA analysis confirmed that the CTB-BC_2_ and CTF-BC_2_ families segregated 1:1 (CT positive vs. negative), with an average of 47.2 and 51.1% of CT-positive seedlings, respectively (*p* ≥ 0.54; Figure 3A). However, the CTG-BC_2_ families deviated from the expected frequency with an average of 61.5% CT positive seedlings (*p* < 0.001, Figure 3A).

A single BC_1_ x BC_1_ pairwise cross (Figure 2) was performed within each event to produce T_2_ progeny and to confirm whether the resulting T_2_ progeny segregated 3:1 for CT positive vs. negative seedlings (i.e., were theoretically 25% homozygous, 50% hemizygous, and 25% null). The BC_1_ genotypes in this cross were selected based on soluble leaf CTs > 1.5% and plant vigor. The leaf CT trait in the CTB-T_2_ and CTF-T_2_ families again segregated according to expected (3:1) frequencies [79.2% (*P* = 0.51) and 78.7% (*P* = 0.21), respectively], while, in the CTG-T_2_ family, 98.1% of the progeny were CT positive, a deviation from the expected frequency (*p* < 0.001, Figure 3A).

### Quantification of Condensed Tannins in Different Generations and the Influence of *TaMYB14-1* Gene Dosage on Condensed Tannin Accumulation in Leaves

Soluble CTs in leaves from the BC_1_, BC_2_, and T_2_ generations described above were quantified to determine the impact of backcrossing and *TaMYB14-1* zygosity on CT accumulation. Only leaves from seedlings with a DMACA score of ≥ 4 were assayed for CTs and included 186, 125, and 186 individuals from the CTB-BC_1_, CTF-BC_1_, and CTG-BC_1_ families, respectively (Figure 3B). Ten null segregant controls per event were also assayed for CT accumulation. Soluble CTs, expressed as % of DM in epigallocatechin equivalents, averaged 0.6% of DM in progeny from CTB-BC_1_, which was significantly lower (*p* < 0.05) than CTs in CTF-BC_1_ and CTG-BC_1_ progeny, which both averaged 0.8% of DM (Figure 3B). Outliers that produced markedly higher CTs than the average were detected in each event [e.g., 2.0% DM (CTG-BC_1_), 1.8% DM (CTB-BC_1_), and 1.6% DM (CTF-BC_1_)] (Figure 3B). In the BC_2_ generation, out of 1,957 seedlings tested, 108, 163, and 243 plants from CTB-BC_2_, CTF-BC_2_, and CTG-BC_2_, respectively, had DMACA scores ≥4 and were assayed for soluble CTs. CTs in CTB-BC_2_ and CTG-BC_2_ averaged 0.6% and were significantly lower (*p* ≤ 0.05) than CTF-BC_2_, which had a mean of 0.8% DM. Outliers were again noted in CTG-BC_2_ and CTF-BC_2_ with soluble CTs of 2 and 1.6%, respectively (Figure 3C). In the T_2_ generation, the mean soluble CTs in CTB-T_2_ and CTG-T_2_ were affected by zygosity. The individuals with 2 copies of the *TaMYB14-1* gene (homozygotes) had significantly higher mean CTs (1.6 and 1.9% DM for CTB-T_2_ and CTG-T_2_, respectively) compared to those with only a single copy (hemizygotes) of the *TaMYB14-1* gene (0.9 and 1% DM for CTB-T_2_ and CTG-T_2_, respectively). This was not observed in CTF-T_2_ where the mean CTs for the hemizygotes and homozygotes were not significantly different (both 0.9% DW, Figure 3D).

### Structure and Composition of Soluble Condensed Tannins in Transgenic White Clover Leaves

**FIGURE 4:**
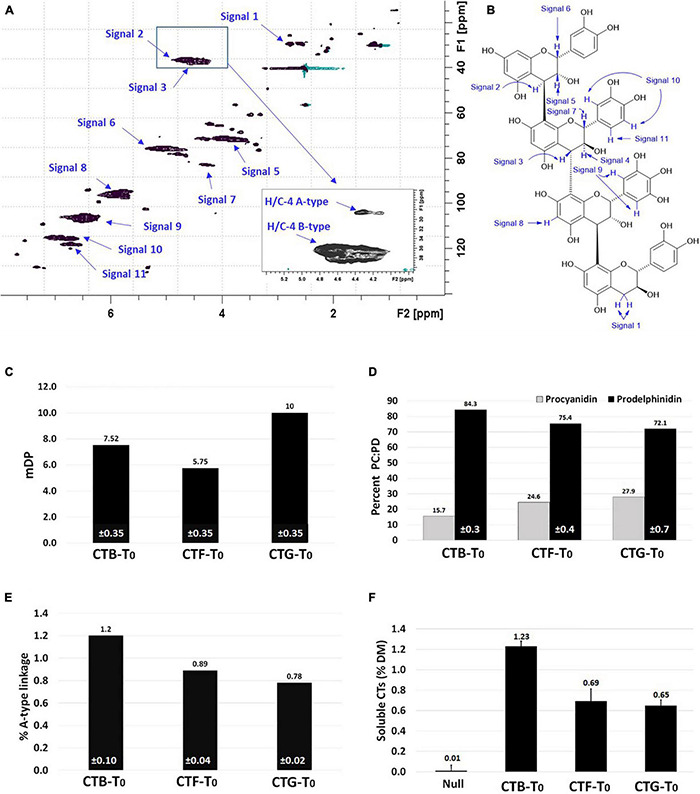
Composition of purified CTs from the leaves of three primary transgenic events. **(A)** A representative ^1^H-^13^C HSQC NMR spectrum of soluble condensed tannins (CTs) from a primary transgenic event (CTB-T_0_) with **(B)** assigned cross-peaks; **(C)** a mean degree of polymerization (mDP); **(D)** the proportion of procyanidin to prodelphinidin (PC:PD) in leaf CTs; **(E)** percent A-type linkage in CTs from three primary events, and **(F)** soluble CTs in the 3 primary events expressed as percent dry matter (DM) of epigallocatechin (EGC) equivalents. The inserted box in panel **(A)** shows an expanded and higher intensity view of the H/C-4 A-type and H/C-4 B-type cross-peaks present in the CTs from CTB-T_0_. The comparative integration of these cross-peaks allows for the proportion of A-type linkages present to be determined. Similarly, the additional composition of the purified CTs was determined through the integration of respective cross-peak volumes (Zeller et al., 2015b; Naumann et al., 2018). The ± values at the base of each bar in panels **(C–E)** are standard deviations from three separate measurements of the same spectrum. Error bars in panel **(F)** represent the standard error of the mean of soluble CTs from three independent harvests at 4, 8, and 12 weeks after planting.

The composition, configuration, and mean degree of polymerization (mDP) of CTs affect their capacity to bind and release proteins, and, therefore, their potential to protect and release the protein in the rumen and intestines, respectively. Therefore, the structure of CTs in the leaves of CTB-T_0_, CTF-T_0_, and CTG-T_0_ was determined independently using ^1^H-^13^C HSQC (Heteronuclear Single Quantum Coherence) nuclear magnetic resonance (NMR) spectroscopy as described (Zeller et al., 2015a; Naumann et al., 2018). The ^1^H-^13^C HSQC NMR spectrum of purified CTs from CTB-T_0_ is given in Figure 4A, along with carbon-hydrogen bond cross-peak assignments (Figure 4B). The CTs in the three transformation events have a amDP of 6 to 10 flavan-3-ol subunits (Figure 4C) and are composed predominantly of prodelphinidin (PD) with 15.7–27.9% procyanidin (PC) subunits (Figure 4D). Purified CTs from all the plants tested contained a small amount (0.78–1.2%) of A-type linkages (Figure 4E). The proportion of A-type linkages present was determined through comparative integration of H/C-4 A-type and H/C-4 B-type cross-peaks (see the inserted box in Figure 4A) in the ^1^H-^13^C HSQC NMR spectrum. The soluble CTs in these transformation events ranged from 0.6% to 1.2% (Figure 4F).

**FIGURE 5:**
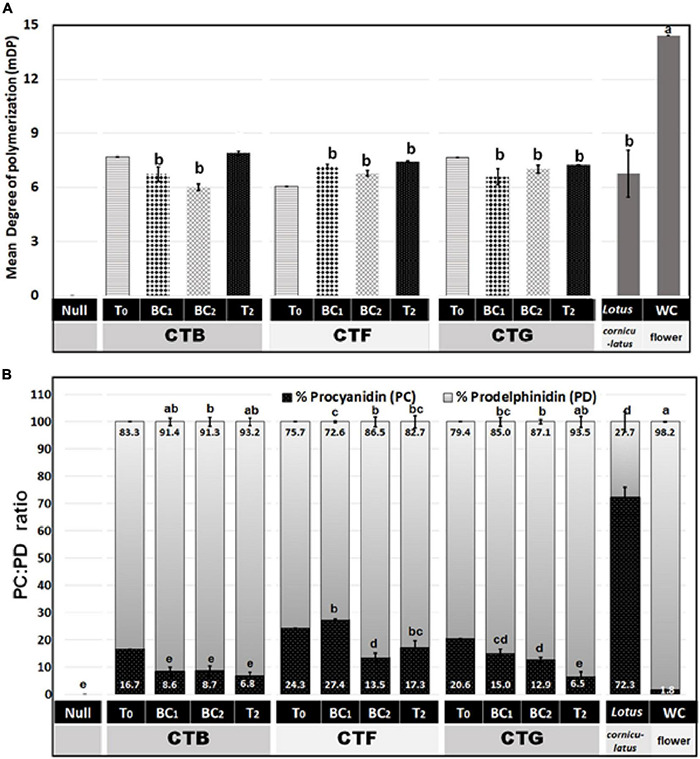
**(A)** A mean degree of polymerization (mDP) and **(B)** percent procyanidin (PC) and prodelphinidin (PD) in white clover CTs from three primary transgenic events (T_0_) plus progenies (at least three independent genotypes per group) from the BC_1_, BC_2_, and T_2_ generations. The PC, PD, and mDP were determined by Waters Xevo UPLC-DAD-MS/MS. Mean comparison was done across BC_1_, BC_2_, and T_2_ data. The T_0_ events were excluded in the mean comparison as there were no biological replicates (only one transformant per event). The *Lotus corniculatus* leaves and white clover inflorescences were provided as a native condensed tannin comparison. In both panels, different letters on top of the bar indicate significant differences among BC_1_, BC_2_, and T_2_ means by Fisher’s Tukey’s Multiple Comparison Test (MCT) *P* ≤ 0.01.

**FIGURE 6:**
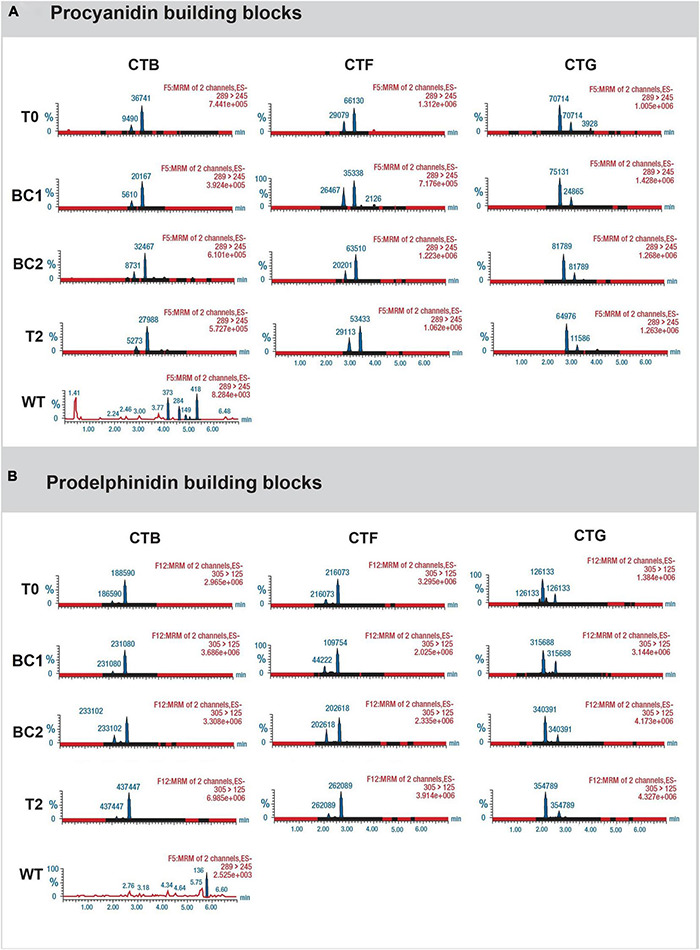
Chromatograms showing **(A)** the presence of catechin and epicatechin (the monomeric PC building blocks), and **(B)** gallocatechin and epigallocatechin (the monomeric PD building blocks) from representative progeny from each generation of the 3 white clover transformation events CTB, CTF, and CTG. Analysis was conducted using the Waters triple quadrupole UPLC-DAD-MS/MS system. For wild-type (WT) samples, the levels were negligible.

To determine whether leaf CT composition was consistent between generations, representative plants from T_0_, BC_1_, BC_2_, and T_2_ were analyzed by UPLC-DAD, coupled with a Xevo triple quadrupole MS instrument where each genotype was treated as a biological replicate for each generation except for the 3 primary T_0_ plants. Samples from wild-type white clover inflorescences (cv. “Grasslands Mainstay”) were also included as a comparator. In this analysis, leaf CTs in the T_0_ generation had mDPs of between 6 and 8 units, comparable to CTs in *L. corniculatus* (7 units), but shorter than CTs from white clover inflorescences (mDP = 14, Figure 5A). The mDP in CTs from intergenerational plants remained stable. Leaf CTs had 73–93% PD and 7–27 % PC, with mean values of 15% PC and 85% PD, giving a PC:PD ratio of 15:85. By contrast, the *L. corniculatus* control had a PC:PD ratio of 72:28 (Figure 5B). The fingerprints of monomeric flavan-3-ol building blocks of PCs (catechin and epicatechin) and PDs (gallocatechin and epigallocatechin) in the CTB, CTF, and CTG remained consistent across generations (Figures 6A,B).

### Protein Binding and Dissociation by White Clover Condensed Tannins

To determine whether white clover leaf CTs can bind and release protein, residual protein concentrations were measured in the supernatant and pellet after the addition of CTs at pH 6.5 and 2.5 or 2.0 (dependent on the protein source). CTs were purified by Sephadex LH-20 chromatography from the leaves of the three T_0_ events (CTB-T_0_, CTF-T_0_, and CTG-T_0_) and were treated as biological replicates. CT concentration was optimized to precipitate 1 mg/ml of protein by testing the effects of 0.3, 0.6, and 1 mg/ml of white clover CTs on bovine serum albumin (BSA) and white clover leaf proteins. The concentrations used were based on a previous study, which showed that 0.8 mg/ml of alfalfa CTs were sufficient to precipitate nearly 100% of leaf protein from solution (Zeller et al., 2015b). Protein precipitation at pH 6.5 was proportional to the concentration of CTs in solution (*R*^2^≥ 0.83 and ≥0.96 for the supernatant and pellet, respectively; Supplementary Figures 5A,B). In the negative controls (no CTs), an average of 85.8% of BSA and 88.2% of white clover protein was detected in the supernatant, and no protein was detected in the pellet. When 1 mg/ml of CTs was added, 77.5 and 74.9% of the BSA and white clover protein, respectively, were detected in the pellet (Supplementary Figures 5A,B).

**FIGURE 7:**
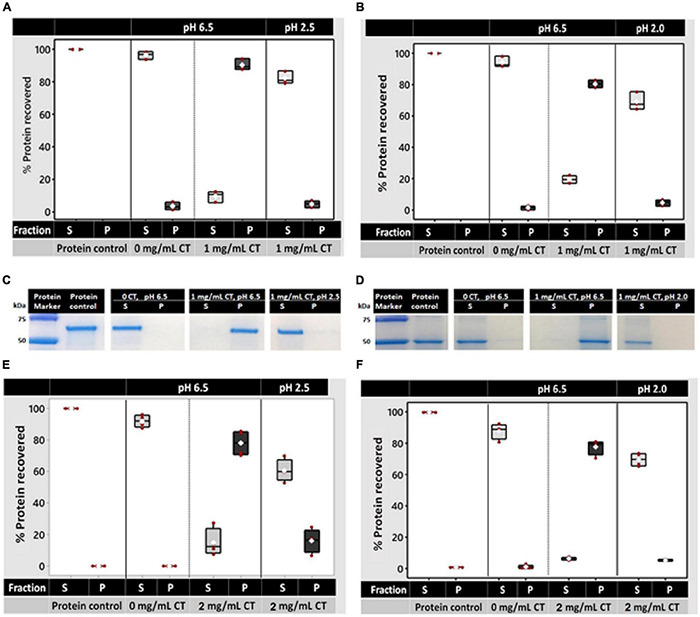
Protein binding and dissociation by white clover leaf CTs and their effects on protein degradation in rumen fluid *in vitro*. CTs used in protein binding and dissociation studies were either purified by column chromatography **(A–D)** or used as crude CT extracts from white clover leaves **(E,F)**. Protein binding was determined by quantifying CT-bound protein in the pellet and residual protein in the supernatant using a Qubit fluorometer (Invitrogen). **(A,B)** Protein binding by CTs (1 mg/ml) at pH 6.5 (rumen pH) and dissociation at pH 2.5 (for BSA) or pH 2.0 (for white clover protein; abomasum pH). **(C,D)** Representative SDS-PAGE gel showing protein partitioning between the supernatant or pellet after 20 min of incubation with 1 mg/ml of column-purified white clover leaf CTs from CTG-T_0_. The protein marker was the Precision Plus Protein Standard™ (Bio-Rad). **(E,F)** Protein binding by crude CT extracts (2 mg/ml) at pH 6.5 and dissociation at pH 2.5 or pH 2.0. In panels **(A–D)**, purified CTs from CTB-T_0_, CTF-T_0_, and CTG-T_0_ were used as biological replicates. In panels **(E,F)**, four independent CT extractions (two T_2_ plants, each with 2 clones) served as biological replicates. Each data point in panels **(A,B,E,F)** represents a biological replicate.

The effect of pH on binding (at pH 6.5) and dissociation at pH 2.5 (for BSA) and pH 2.0 (for white clover protein) with 1 mg/ml CT was then determined. A different dissociation pH for the two proteins used in pilot studies showed that white clover protein did not dissociate at pH 2.5. In control solutions (no CTs) of pH 6.5, > 96% of both proteins was found in the supernatant after incubation. After the incubation with 1 mg/ml CTs, 90.4 and 80.5% of BSA and white clover protein, respectively, were recovered in the pellet with 9.5–19.5% detected in the supernatant (Figures 7A,B). To determine whether the CT-protein complex dissociated in the acidic pH of the abomasum, the protein-CT pellets were resuspended in a solution of pH 2.5 (for BSA) or pH 2.0 (for white clover protein), incubated, and recentrifuged to recover any residual protein-CT complexes. Protein assays indicated that 82.3% of the BSA and 69.2% of the white clover protein had disassociated from the CTs and were detected in the supernatant (Figures 7A,B). Sodium dodecyl sulfate-polyacrylamide gel electrophoresis (SDS-PAGE) provided visual confirmation of protein partitioning between the supernatant and pellet and confirmed that they had remained intact, ∼66 kDa and ∼50 kDa for BSA and white clover, respectively (Figures 7C,D).

To determine whether crude CT extracts from white clover leaves were also effective in binding protein, soluble CTs from two homozygous CTG-T_2_ plants (3755 and 3764, two clones of each) were extracted as described in Supplementary Methods “Extraction and Quantification of Soluble and Insoluble CTs in White Clover Leaves,” quantified, freeze-dried, and resuspended in 50 mM of a 2-[N-morpholino]ethanesulfonic acid (MES) buffer at pH 6.5 and tested in the protein precipitation assay as described above. Here, 2 mg/ml of crude CTs was required to precipitate 78.1% of BSA and 77.7% of white clover proteins (1 mg/ml). As above, upon resuspension and incubation of the pellet in pH 2.5 or 2., 60.6% of the BSA and 69.5% of white clover proteins, respectively, were recovered in the supernatant after centrifugation (Figures 7E,F).

### Effects of White Clover Leaf Condensed Tannins on Ammonia and Volatile Fatty Acid Production *in vitro*

The impact of white clover CTs on protein degradation in rumen fluid was tested *in vitro* in a custom-designed array of sealed anaerobic incubation flasks. The test substrates were freeze-dried leaf powder from two independent homozygous CTG-T_2_ genotypes (3755 and 3764), plus non-transformed (WT) white clover inflorescences and leaves as positive and negative CT controls, respectively. Soluble leaf CTs ranged from 1.6 to 2.4% of DM and were composed of 85.1–84.2% PD and 14.9–15.8% PC, with an mDP of 10–11 units. The white clover inflorescence sample contained soluble CT concentrations of 1.9% DM and was composed of 98.2% PD and 1.8% PC with an mDP of 15 units (Supplementary Table 3). The nutrient composition is presented in Supplementary Table 4. To account for potential substrate composition effects, protein degradation products in rumen fluid were compared in duplicate fermentations for each sample, one with and one without polyethylene glycol (PEG) 6000. PEG binds with high affinity to CTs (Wang et al., 1996; Besharati and Taghizadeh, 2011), which effectively removes all expected CT influences on protein fermentation. The difference in ammonia and volatile fatty acid concentration in flasks with and without PEG 6000 was used to estimate the specific contribution of CTs to protein protection (Table 1). The experiment was repeated three times using rumen fluid from two cows each time (six cows in total). After 6 h of incubation in leaf powders from genotype 3755 and 3764 without PEG, ammonia production decreased by 60 and 45%, isovalerate by 5 and 38%, and isobutyrate by 30 and 24% (*p* ≤ 0.01) relative to the leaves of WT negative control, respectively (Table 1). In fermentations containing white clover inflorescences (positive control), ammonia and isovaleric acid concentrations were lowered by 24 and 74%, respectively. Control WT white clover leaves had no detectable reduction in ammonia or isovaleric acid concentration at either time. After 24 h of incubation, ammonia, isovalerate, and isobutyrate production in CT-containing substrates were still significantly reduced (*p* ≤ 0.05) relative to the WT white clover leaves (Table 1).

**TABLE 1:** Production of ammonia, isovalerate, and isobutyrate after 6 and 24 h of incubation in rumen fluid with and without polyethylene glycol 6000 (PEG) *in vitro*^§^.

Variable	Substrate	+ PEG		− PEG		% reduction^1^ (due to PEG)	% reduction relative to WT WC lvs^2^
		(*N* = 6)		(*N* = 6)			
		Mean	*SD*	Mean	*SD*		
**6 h Incubation**							
NH4	3755	164.4	36.6	64.7	19.1	60.6^a^	60.5
(nmol/g)	3764	149.2	45.3	81.4	36.2	45.4^ab^	45.3
	WT WC Infl	117.2	35.8	89.0	31.3	24.1^b^	23.9
	WT WC lvs	83.1	39.3	83.0	41.5	0.1*^c^*	0.0
Isovalerate	3755	77.7	11.6	36.5	5.8	53.0^b^	52.0
(nmol/g)	3764	83.7	16.2	52.2	11.9	37.7*^c^*	36.7
	WT WC Infl	43.2	10.5	11.2	2.0	74.1^a^	73.1
	WT WC lvs	53.6	10.9	54.1	9.2	1.0^a^	0.0
Isobutyrate	3755	34.2	4.7	24.0	6.0	29.8^b^	27.5
(nmol/g)	3764	36.5	7.7	27.9	7.2	23.6^b^	21.3
	WT WC Infl	27.3	5.4	12.0	2.5	55.9^a^	53.6
	WT WC lvs	21.9	6.0	22.4	5.5	2.3^c^	0.0
**24 h incubation**							
NH4	3755	367.4	13.5	278.0	37.1	24.3^a^	23.2
(nmol/g)	3764	368.3	47.3	325.8	49.4	11.5^a^	10.4
	WT WC Infl	272.9	33.7	221.6	19.1	18.8^a^	17.7
	WT WC lvs	295.0	42.0	298.2	24.2	1.1^b^	0.0
Isovalerate	3755	179.1	29.6	125.7	20.7	29.8^a^	27.9
(nmol/g)	3764	183.6	34.9	148.2	29.6	19.3^b^	17.4
	WT WC Infl	115.4	21.5	73.0	13.0	36.8^a^	34.9
	WT WC lvs	153.4	23.3	156.3	20.9	1.9*^c^*	0.0
Isobutyrate	3755	98.38	15.64	71.54	12.36	27.28^a^	25.96
(nmol/g)	3764	101.99	18.03	82.96	17.80	18.66^a^	17.33
	WT WC Infl	63.34	9.96	44.97	6.40	29.00^a^	27.68
	WT WC lvs	87.59	11.30	88.75	10.87	1.32^b^	0.00

*^§^ Effects of CTs are expressed as percent reduction due to PEG 6000 (which complexes with CTs) and percent reduction relative to control (WT WC leaves)*

*^1^Within each variable, means that do not share a letter are significantly different at p ≤ 0.01 using Tukey’s Multiple Comparison Test (MCT).*

*^2^Percent reduction relative to wild type control was calculated using the values in the column for % reduction due to the effects of PEG.*

*SD, standard deviation from the mean; WT WC, wild-type white clover; Infl, inflorescence; lvs, leaves.*

The concentration of propionate and butyrate in leaf powder from CT-containing genotypes was also reduced by an average of 19.3 and 16.9% (*p* ≤ 0.01), respectively, after 6 h of incubation, but the production of acetate was not affected (Table 2). Furthermore, the CT-containing substrates promoted a higher acetate to a propionate ratio relative to the WT control.

**TABLE 2:** Production of predominant short chain fatty acids (SCFA) after 6 h and 24 h of incubation in rumen fluid with and without polyethylene glycol 6000 (PEG) *in vitro*^§^.

Variable	Substrate	+ PEG		−PEG		% reduction^1^ due to PEG	% reduction relative to WT WC lvs^2^
		(*N* = 6)		(*N* = 6)			
		Mean	*SD*	Mean	*SD*		
**6 h incubation**							
Acetate	3755	2.49	0.14	2.26	0.2	9.11^ab^	13.88
(nmol/g)	3764	2.47	0.14	2.28	0.16	7.65^ab^	12.42
	WT WC Infl	2.75	0.35	2.23	0.42	18.87^a^	23.64
	WT WC lvs	2.27	0.39	2.37	0.33	−4.77^b^	0.00
Propionate	3755	0.59	0.05	0.43	0.06	27.1^a^	23.99
(nmol/g)	3764	0.62	0.08	0.51	0.07	17.7^a^	14.62
	WT WC Infl	0.78	0.13	0.61	0.13	21.8^a^	18.67
	WT WC lvs	0.64	0.13	0.66	0.13	3.1^b^	0.00
Butyrate	3755	0.45	0.02	0.37	0.02	16.4^a^	19.01
(nmol/g)	3764	0.46	0.03	0.40	0.03	12.3^a^	14.91
	WT WC Infl	0.45	0.04	0.34	0.04	23.5^a^	26.12
	WT WC lvs	0.50	0.05	0.51	0.05	2.6^b^	0.00
A:P	3755	4.27	0.36	5.31	0.48		
	3764	4.06	0.33	4.53	0.38		
	WT WC Infl	3.54	0.38	3.65	0.23		
	WT WC lvs	3.54	0.26	3.63	0.28		
**24 h incubation**							
Acetate	3755	3.31	0.24	3.25	0.23	1.8^a^	5.42
(nmol/g)	3764	3.23	0.28	3.24	0.32	−0.3^ab^	3.29
	WT WC Infl	3.67	0.15	3.57	0.16	2.7^a^	6.33
	WT WC lvs	3.33	0.18	3.45	0.23	−3.6^b^	0.00
Propionate	3755	0.72	0.12	0.61	0.1	15.3^a^	16.47
(nmol/g)	3764	0.74	0.13	0.69	0.12	6.7^b^	7.95
	WT WC Infl	0.92	0.14	0.83	0.11	9.8^a^	10.97
	WT WC lvs	0.84	0.12	0.85	0.12	−1.2*^c^*	0.00
Butyrate	3755	0.63	0.06	0.60	0.04	4.4^ab^	6.78
(nmol/g)	3764	0.64	0.06	0.63	0.07	2.2^ab^	4.60
	WT WC Infl	0.70	0.02	0.65	0.01	7.2^a^	9.59
	WT WC lvs	0.80	0.03	0.82	0.03	2.3^b^	0.00
A:P	3755	4.65	0.40	5.40	0.48		
	3764	4.45	0.37	4.77	0.35		
	WT WC Infl	4.04	0.42	4.34	0.40		
	WT WC lvs	4.01	0.37	4.07	0.37		

*^§^ Effects of CTs are expressed as percent reduction due to PEG 6000 (which complexes with CTs) and percent reduction relative to control (WT WC leaves).*

*^1^Within each variable, means that do not share a letter are significantly different at p ≤ 0.01 using Tukey’s Multiple Comparison Test (MCT).*

*^2^Percent reduction relative to wild type control was calculated using the values in the column for % reduction due to the effects of PEG.*

*SD, standard deviation from the mean; WT WC, wild-type white clover; Infl, inflorescence; lvs, leaves.*

### White Clover Leaf Condensed Tannins Reduce Methane Emissions Using *in vitro* Rumen Fluid

The potential for white clover leaf CTs to reduce methane emissions was determined in an automated batch culture system (Muetzel et al., 2014). The substrates were identical to those described in the preceding section. Control WT white clover leaves had negligible impacts on total gas and methane production (-2.6 and 1.7%, respectively), while the leaf CTs reduced total gasses by an average of 14.8 and 8.2% and methane by 17.1 and 19.4% for genotypes 3755 and 3764, respectively, after 6 h (Table 3). White clover inflorescence similarly reduced total gasses and methane by 17.1 and 20.2%, respectively. After 24 h, when reaction conditions were being constrained by substrate limitation, mean reductions in total gasses were 6.1 and 5.2%, and, for methane, were 9.8 and 9% for genotype 3755 and 3764 leaves, respectively (Table 3). The overall effects of CT-containing leaves were a reduction in total gas of 11.5 and 5.6 at 6 and 24 h, and methane emissions of 18.2 and 9.3% at 6 and 24 h, respectively (Figure 7B), both reductions were significantly greater than control WT white clover leaves (*p* ≤ 0.01).

**TABLE 3:** Methane and total gas production after 6 h and 24 h of incubation in rumen fluid with and without polyethylene glycol 6000 (PEG) *in vitro*^§^.

Variable	Substrate	+ PEG		− PEG		% reduction^1^ (due to PEG)	% reduction relative to WT WC lvs^2^
		(*N* = 6)		(*N* = 6)			
		Mean	*SD*	Mean	*SD*		
**6 h incubation**							
CH4	3755	20.6	1.1	17.1	0.7	17.1^a^	15.4
(mL/g)	3764	22.6	1.1	18.2	1.0	19.4^a^	17.7
	WT WC Infl	26.7	1.8	21.3	2.0	20.2^a^	18.6
	WT WC lvs	20.5	1.9	20.1	1.8	1.7^b^	0.0
Gas	3755	188.7	9.8	160.7	9.9	14.8^ab^	12.2
production	3764	189.1	13.1	173.5	5.4	8.2^b^	5.7
(mL/g)	WT WC Infl	217.9	11.3	180.6	13.0	17.1^a^	14.5
	WT WC lvs	193.3	11.3	198.3	9.4	2.6^a^	0.0
**24 h incubation**							
CH4	3755	40.6	0.2	36.6	1.2	9.8^a^	9.1
(mL/g)	3764	44.7	0.3	40.7	0.6	9.0^a^	8.3
	WT WC Infl	50.6	1.1	47.3	0.5	6.5^b^	5.9
	WT WC lvs	45.7	1.1	46.5	0.2	0.6*^c^*	0.0
Gas	3755	276.2	4.3	259.3	10.3	6.1^a^	5.4
production	3764	284.7	1.2	270.0	5.2	5.2^a^	4.4
(mL/g)	WT WC Infl	315.9	3.3	297.7	4.5	5.8^a^	5.0
	WT WC lvs	301.1	1.5	303.4	2.5	0.7^b^	0.0

*^§^Effects of CTs are expressed as percent reduction due to PEG 6000 (which complexes with CTs) and percent reduction relative to control (WT WC leaves).*

*^1^Within each variable, means that do not share a letter are significantly different at p ≤ 0.01 using Tukey’s Multiple Comparison Test (MCT).*

*^2^Percent reduction relative to wild-type control was calculated using the values in the column for % reduction due to the effects of PEG.*

*SD, standard deviation from the mean; WT WC, wild-type white clover; Infl, inflorescence; lvs, leaves.*

## Discussion

A high-performing white clover cultivar “Grasslands Mainstay” has been transformed with the MYB transcription factor *TaMYB14-1*, resulting in stable CT accumulation in leaves across three generations (T_0_, BC_1_, and BC_2_/T_2_). Accumulation of CTs was influenced by gene dosage and significantly elevated in homozygous T_2_ progeny. The levels of CTs in these plants were effective in binding protein at the ruminal pH (pH 6.5) and were able to dissociate at the pH of the abomasum (pH 2.5 for BSA and pH 2.0 for white clover protein). CT-containing white clover leaves also reduced ammonia and methane production when tested in the rumen *in vitro* assays, indicating that these plants have the potential to reduce N excretion and methane emissions from ruminants.

### White Clover Transformation and Enhancement of Condensed Tannins in Leaves Through Gene Dosage

Stable inheritance is improved by having a single gene insert (Tang et al., 2007). Hence, to generate plants with a stable CT trait, three different transformation events were selected, containing a single insertion of the *TaMYB14-1* gene that produced moderate leaf CT concentrations (0.77–1.28% DM; Supplementary Figures 1E, 3B,**C**). Furthermore, since redundant vector backbone sequences beyond the T-DNA borders are undesirable for biotechnology applications (Ye et al., 2008; Wang et al., 2016), these sequences were confirmed as being absent (Supplementary Figure 4) in plants before using them in plant breeding.

White clover is an obligate outcrossing species with a multi-allelic self-incompatibility system (Casey et al., 2010). The CT trait in the BC_1_, BC_2_, and T_2_ progeny followed the expected patterns of segregation in the progeny (1:1 and 3:1 positive and null segregants in the BC and T_2_ generation, respectively (Figure 3A), except for the CTG transformant where the observed CT positive individuals in BC_2_ and T_2_ progeny were higher than expected (Figure 3A). Retrospective zygosity testing confirmed that this was due to a probable selfing event, which produced a homozygous BC_1_ plant that was used in later pairwise and backcrosses. Self-fertilization is not a common occurrence in white clover but can be induced by high temperatures (Douglas and Connolly, 1989).

Condensed tannin accumulation in leaves of plants in the two successive BC generations remained stable for the three transformation events, indicating that selection and breeding did not enhance or decrease CTs but maintained their levels in each generation. However, the pairwise crosses showed that gene dosage elevated CTs in leaves approximately twofold in homozygous versus hemizygous progeny of the CTB-T_2_ and CTG-T_2_ generation (Figure 3D). *TaMYB14-1* homozygosity may, therefore, be an essential strategy for maintaining CTs levels in white clover leaves during breeding. Gene dosage effects have been reported to be positively correlated with gene expression and recombinant protein application in maize (Hood et al., 2012) and in some genes in *Brassica* polyploids and their hybrids (Tan et al., 2016). Since MYB transcription factors are key players in engineering CT synthesis in plants (Dixon et al., 2013) and that MYB14 may form complexes with other transcription factors, it is plausible that the two copies of the *TaMYB14-1* gene form a ternary complex with other transcription factors, resulting in significantly higher levels of CTs in white clover leaves. Surprisingly, in CTF-T_2_, the accumulation of CTs was comparable in hemizygous and homozygous progeny (Figure 3D). Factors, such as chromosomal effects on the integration site, or changes in the methylation status of regulatory elements of the transgene, have been reported to affect gene expression (Feng et al., 2001; Chanda et al., 2017) and could result in unexpected transcription and associated processes.

### Composition of Condensed Tannins in Transgenic Clover Leaves

The use of ^1^H-^13^C HSQC NMR spectroscopy is a relatively new technique for the determination of CT structure and composition. Comparison of the integrated volumes of respective NMR cross-peaks signals in Figure 4A allowed for estimation of the procyanidin to prodelphinidin (PC/PD) ratio (volume of signal 11/one-half the volume of signal 9) and the mean degree of polymerization [volumes of signals 1 × 0.694 (correction factor), 2, and 3/volume of signal 1 × 0.694]. Values obtained using these methods have been shown to strongly correlate with values obtained from thiolytic degradation studies. The PC/PD ratios and mDP are consistent across all three (T_0_) transgenic events.

### Protein Binding by White Clover Condensed Tannins

Protein precipitation (a proxy for protein binding) at pH 6.5 was highly positively correlated with white clover leaf CT concentration, with an *R*^2^ ≥ 0.94 and ≥ 0.96 for BSA and white clover proteins, respectively (Supplementary Figures 5A,B). This observation conforms with previous findings that CT concentration in solution correlates with protein precipitation (Naumann et al., 2014). Furthermore, white clover leaf CTs are rich in PD (73–93%) compared to PC (7–27%, Figure 5B). The additional hydroxyl group on the β-ring of PD flavan-3-ol subunits increases the probability of hydrogen bond formation, resulting in stronger interactions with proteins (Haslam, 1996; Leppä et al., 2020), and this may also contribute to effective CT-protein complexation and precipitation.

The CT-protein complex must be able to dissociate from the CTs in the acidic abomasum (pH 2–3) (Dentinho and Bessa, 2016) to allow for protein digestion and amino acid absorption (McMahon et al., 2000; Naumann et al., 2017). Resuspending the CT-bound protein pellet in an acidic solution (pH 2.5 or 2.0 for BSA or white clover protein, respectively), resulted in the CT-protein complex dissociation, with the released protein again detected in the supernatant (Figures 7A,B). A lower pH of 2.0 was required to release the white clover protein from the CT-protein complex relative to that of the BSA protein. It is highly probable that this was due to differences in protein structure, which may affect the pH required for dissociation. Different proteins vary in pH optima and, depending on the protein, may have a narrow or wide pH range for binding with CTs. A narrow pH range for CT binding is likely to result in the rapid release of most CTs within a small pH change. For BSA, the CT-binding pH range may be narrow, hence liberating CTs easier than other types of proteins, such as white clover proteins. Differences in protein behavior have been reported by Dentinho and Bessa (2016), who demonstrated that rock rose and grape seed CTs precipitate soybean meal protein at pH 6–8, with the CT-protein complex dissociating at pH 2.0. The observed CT-protein binding at ruminal pH supports the proposition that white clover leaf CTs may be able to reduce the degradation of dietary protein in the rumen by microbes. CT dissociation from proteins at pH 2.5 or 2.0 suggests that the proteins could be released in the abomasum, which could lead to improved animal performance (Waghorn et al., 1990; McNabb et al., 1993; Koenig and Beauchemin, 2018). SDS-PAGE assays in the current study showed that the proteins were recovered intact at their expected sizes [∼66 kDa for BSA and ∼50 kDa for white clover protein, which was mainly comprised of the RUBISCO large subunit (Figures 7C,D), respectively]. These observations confirmed that the proteins did not degrade after incubation at pH 6.5, pH 2.5, or pH 2.0 and that the CT-protein complex had disassociated in the low pH buffers as the proteins were the same size as the unbound controls.

Since protein protection assays in rumen fluid are typically confounded by microbial protein, CT-containing white clover leaves were investigated for their ability to protect protein by measuring end products of proteolysis and deamination by ruminal bacteria, including ammonia and isovaleric acids and other VFA (Eschenlauer et al., 2002; Hassanat and Benchaar, 2013; Grosse Brinkhaus et al., 2017). In rumen studies, polyethlene glycol (PEG) MW6000 is used to bind tannins and create a negative control for each tannin treatment that is not affected by nutrient composition (Silanikove et al., 1996). CT-containing white clover inflorescences and leaves markedly reduced ammonia, isovalerate, isobutyrate, propionate, and butyrate production relative to WT white clover leaves (Tables 1, 2). The plant with the highest leaf CTs, genotype 3755 (2.4% DM soluble CTs), was more effective than white clover inflorescences (1.9% DM soluble CTs) in reducing ammonia production in rumen fluid after 6 h (60.6 and 24%, respectively; Table 1). After 24 h of incubation, the substrate in the incubation is essentially depleted, and the ammonia results were increasingly affected by microbial lysis (Vaga and Huhtanen, 2018). Nevertheless, ammonia, isovaleric acid, and other SCFA were still significantly reduced (*p* ≤ 0.01) in all samples containing CTs relative to the control (Tables 1, 2), further confirming the protective role of CTs in rumen fluid *in vivo*. These results are consistent with previous findings that CT-containing sainfoin significantly reduced ammonia, acetate, propionate, and butyrate production when used in *in vitro* fermentation (Theodoridou et al., 2011; Bueno et al., 2020). The reduction in protein degradation in the rumen is due to the formation of CT-protein complexes (Mueller-Harvey, 2006), which are resistant to microbial degradation (McSweeney et al., 2001; Bueno et al., 2020). The lower SCFA production and higher acetate to the propionate ratio further suggest inhibitory effects of CTs on organic matter degradability in the rumen, which improves ruminant nutrition (Hervas et al., 2000; Patra and Saxena, 2011). This could be an additional benefit, considering that the crude protein (CP) in white clover leaves used as substrates ranged from 26.5 to 29.6% (w/w) of DM (Supplementary Table 4), which exceed the required dietary CP concentrations for animals, grazing temperate forages. CP concentrations exceeding 20% of DM are surplus to requirements and can have negative impacts both on animal welfare and the environment (Pacheco and Waghorn, 2008).

Approximately, 70–80% of dietary protein is estimated to be degraded and deaminated by rumen microbes, and 25–35% of the N lost is in the form of ammonia (Barry and McNabb, 1999; Koenig and Beauchemin, 2018), a major nutritional inefficiency in ruminants (Eschenlauer et al., 2002) and a cause of N leaching into waterways (Dymond et al., 2013). Collectively, these data suggest that white clover CT expression in leaves has the potential to confer the proposed benefits to animal nutrition (McNabb et al., 1993; Naumann et al., 2017; Kelln et al., 2021) and supports previous claims concerning the benefit of CTs from sainfoin (*Onobrychis* spp.) (Theodoridou et al., 2012), *Lotus* (Tavendale et al., 2005; Mueller-Harvey et al., 2019), and other CT-containing legumes (Patra and Saxena, 2010; Hassanat and Benchaar, 2013).

### Effects of Condensed Tannins on Methane Production in Rumen Fluid *in vitro*

Leaves of two transgenic white clover genotypes (3755 and 3764) were compared with a non-transgenic (HS132/9 R1) control, as well as CT-producing WT white clover inflorescences, to determine the effects of CTs on total gas and methane emissions in rumen fluid *in vitro*. Total CTs in the leaves of genotypes 3755 and 3764 and WT white clover flowers ranged from 4.9 to 5.6% of DM and soluble CT concentrations ranged from 1.6 to 2.4% of DM (Supplementary Table 3). The greatest impact of leaf CTs occurred at 6 h of incubation, where reductions in total gas and methane emissions averaged 11.5 and 18.2% (*p* ≤ 0.01), respectively, for transgenic plants 3755 and 3764. This indicates that, although CTs have a slightly negative effect on total fermentation, the effect on methane production is pronounced. Conversely, no effects on methane production were detected when WT control leaves were used. The effect of CTs in the current study is relatively greater than previous *in vitro* fermentation studies where the inclusion of 50 g kg^–1^acacia tannin extracts reduced methane production by 12% (*p* ≤ 0.01), but lower than the effects of 100 g kg^–1^or 200 g kg^–1^quebracho tannin extracts, which reduced methane by 23 and 40% (*p* ≤ 0.01), respectively, relative to control (Hassanat and Benchaar, 2013). Reduction in methane by up to 27% relative to control (*p* ≤ 0.01) was also achieved in an i*n vitro* fermentation using sainfoin leaves (Theodoridou et al., 2011), suggesting the possibility that a greater reduction in methane is potentially achievable with white clover leaves, containing a higher level of CT.

Methanogenesis is affected by several factors, such as differences in DM digestibility and fiber content (Waghorn et al., 2002; Danielsson et al., 2017), as well as CT quantity and composition (Mueller-Harvey et al., 2019; Min et al., 2020). Since the leaf substrates used were grown and harvested at similar growth stages, had similar nutritional composition (Supplementary Table 4), and matrix effects were controlled using PEG 6000, the amount and the composition of the CTs are the most likely explanation for the observed reduction in methane production. This confirms previous reports that CTs in ruminant feed reduce methane emissions (Waghorn et al., 2002; Woodward et al., 2004; Tavendale et al., 2005). Additionally, the effects of CTs on methanogenesis also depend on the length of the CT polymers (Waghorn and McNabb, 2003; Mueller-Harvey, 2006). In the *in vitro* rumen fermentation studies, the CTs in leaves of genotypes 3755 and 3764 had an mDP of 10–11 units with a PD:PC of 85:15, while CTs in WT white clover inflorescences had an mDP of 15 units with a PD:PC ratio of 98:2 (Supplementary Table 3). The white clover leaf CT composition is relatively similar to the composition of CTs in sainfoin (*Onobrychisviciifolia*) leaves with mDPs of 11.8 units and prodelphinidin of 76.4%. This chemistry resembles other studies where oligomeric CTs have been shown to have a higher H-bonding strength and were effective against methanogens (Tavendale et al., 2005). Similarly, Baert et al. (2016) reported that both oligomeric and polymeric ellagitannins decreased methane production on ruminal fermentation *in vitro* in a size-dependent manner.

## Summary

This study has demonstrated that the CT trait in the leaves of transformed elite white clover cultivar is stable over at least two generations. CT expression is elevated by gene dosage (homozygosity) to a level (>2% DM) predicted to reduce protein fermentation and methane emissions in animals. White clover leaf CTs were able to reduce methane emissions in rumen fluid *in vitro* by up to 19.4%. A reduction in total gasses is an indication of reduced protein fermentation, which implies that CTs in the feed may also reduce animal productivity through reduced dietary fermentation (Pellikaan et al., 2011). However, this needs to be examined in more detail as changes in fermentation pathways also lead to differences in total gas production. Nevertheless, this potential deficit is outweighed by the net benefits of increased protein uptake in the hindgut, improved environmental (reduced methane and N_2_O emissions), and animal health (reductions in bloat) benefits of white clover containing CTs in leaves. This has implications for using CT expressing white clover for reduced greenhouse gas emissions. Controlled animal trials will be essential for confirming these assumptions.

## Data Availability Statement

The datasets presented in this study can be found in online repositories. The names of the repository/repositories and accession number(s) can be found in the article/Supplementary Material.

## Ethics Statement

The animal study was reviewed and approved by AgResearch Grasslands Animal Ethics Committee Approval #13398.

## Author Contributions

MR, CV, DW, and JC designed the project. GC performed plant breeding with input from DW, JC, and ZJ. WZ, KF, and J-PS analyzed condensed tannin structure and composition. SM and AB contributed to methane studies, white clover transformations by MR, KR, and DM. MR and RK conducted all other experiments. MR performed data analysis in consultation with AgResearch statistical scientist Dongwen Luo (see section “Acknowledgments”). MR, WZ, SM, and CV wrote the manuscript with editing by all other authors. All authors contributed to the article and approved the submitted version.

## Conflict of Interest

JC is employed by Grasslanz Technology Ltd., who owns the patent for the TaMYB14 transcription factor. DW was the primary breeder involved with the development of the cultivar “Grasslands Mainstay” and is employed by PGG Wrightson Seeds Ltd., who will have the exclusive license from Grasslanz Technology Ltd for use of TaMYB14 in white clover. The remaining authors declare that the research was conducted in the absence of any commercial or financial relationships that could be construed as a potential conflict of interest.

## Publisher’s Note

All claims expressed in this article are solely those of the authors and do not necessarily represent those of their affiliated organizations, or those of the publisher, the editors and the reviewers. Any product that may be evaluated in this article, or claim that may be made by its manufacturer, is not guaranteed or endorsed by the publisher.
