# Supplementary material for: Condensed Tannins in White Clover (Trifolium repens) Foliar Tissues Expressing the Transcription Factor TaMYB14-1 Bind to Forage Protein and Reduce Ammonia and Methane Emissions in vitro
Source: Front Plant Sci. 2022 Jan 6;12:777354. doi: 10.3389/fpls.2021.777354 (PMC8774771; doi:10.3389/fpls.2021.777354)
Supplement: Supplementary file 1 [file Table_1.pdf]

Supplementary Material

1. METHODS

1.1 White clover transformation

Transgenic white clover lines were produced by *Agrobacterium*-mediated transformation of cotyledonary explants as previously described (Roldan et al., 2016; Voisey et al., 1994) using *A. tumefaciens* strain GV3101. Putative transformants, which survived kanamycin selection (80 µg/mL), were screened for the expression of condensed tannins in their leaves by a histochemical assay using the chromogenic reagent dimethylaminocinnamaldehyde (DMACA) as described (Li et al., 1996), and the presence of the transgene was confirmed by PCR of genomic DNA using MR183 (forward) and MR184 (reverse) primers (Table S2) which bind to the CaMV35S promoter sequences upstream of the *Ta-MYB14-1* gene (Fig S3A). Vector contamination was determined by PCR of genomic DNA using a panel of primer pair (Table S2) designed to span all regions of the Ti plasmid (Figure S4A), including those sequences flanking the left and right borders. Transgenic plants were maintained under PC2 containment glasshouse conditions with a temperature range usually between 15 and 22°C.

1.2 Determination of *TaMYB14-1* copy number

White clover DNA for regular PCR and droplet digital PCR (ddPCR) analysis was routinely extracted using the Geneaid Genomic DNA Mini Kit (Plant) following the manufacturer's protocol. DNA for Southern blot hybridization was isolated using the Cetyl trimethyl ammonium bromide (CTAB) method (Doyle & Doyle, 1987) and 10 µg DNA was digested with the *Hind*III restriction enzyme. The number of T-DNA insertion events in the primary transformants was first determined by Southern blot hybridisation using a digoxigenin (DIG) - labelled probe homologous to the CaMV35S promoter (Fig S3A). The probe (787 bp) was synthesized from 4 ng of pART27-35S-TaMYB14-1 plasmid DNA using sense (MR183) and antisense (MR184) primers (Table S2). Probe synthesis, blotting, hybridisation and detection were performed as specified in the DIG application manual for filter hybridisation (Roche Diagnostics, Manheim, Germany). Determination of transgene copy number by ddPCR was carried out as described (Bio-Rad, 2017; Mazaika & Homsy, 2014) with modifications. *TaMYB14-1* and the reference gene (TR-ACO1, GenBank: DQ112347.1) (Chen &

McManus, 2006) were amplified from 50 ng of genomic DNA using primers MR252/MR253 and MR254/MR255, respectively (Table S2). Two TaqMan hydrolysis probes (PR7 and PR8, both double quenched, synthesized by Integrated DNA Technologies), with either 5’-FAM or 5’-HEX fluorescent reporter dye and ZEN (internal)- 3′ Iowa Black FQ (BkFQ) quenchers were used in the ddPCR assay. The labelled probe PR7 (5’-/56-FAM/ATG ACG CAC/ZEN/AAT/CCC ACT ATC C/31ABkFQ/-3’) was used to target the gene of interest (GOI), while the labelled probe PR8 (5’-/5HEX/TCT AAT CTC /ZEN/CCT CTG ATC GCA GC/31ABkFQ/ -3’) targets the Tr-ACO1 reference gene. Copy number determination by ddPCR was also routinely used to determine zygosity of individual progenies.

1.3 Plant maintenance

The primary transgenic (T_0) plants were transferred to soil in pots, and progeny were sown directly into soil. Plants were grown and maintained in a PC2 containment glasshouse. All plant breeding activities were performed in this facility with day and night temperature usually within 15 - 22°C. All samples for CT analysis, including those for CT structure and composition analysis and *in vitro* methane and ammonia analyses were collected from glasshouse-grown plants.

1.4 Breeding strategy and segregation analysis

In this study, the wild type elite genotypes (at least 10 per event) of the large leaf white clover cultivar, ‘Grasslands Mainstay’, were manually backcrossed to the CTB-T_0, CTF-T_0 and CTG-T_0 transformation events (Figure 2). To do this, pollen from flowers of transgenic individuals were collected manually and introduced into the stigma of the non-transformed ‘Mainstay’, and vice-versa (in reciprocal crosses). Mature flower heads were harvested a month after pollination, and seeds were cleaned, germinated and sown as described (Roldan et al., 2020) prior to progeny evaluation. In the BC_1 progeny, 12 seed from each reciprocal pairwise cross were sown (24 seed in total). When the first trifoliate leaf open, seedlings were manually scored for CT synthesis by DMACA (scale = 0-7, Figure S2). Plants with a leaflet DMACA score of ≥ 1 were considered positive for the introduced gene (Roldan et al., 2020) and scores were used to estimate the segregation ratio of the gene in the progeny. Plants were also visually scored for vigor [1 (less vigorous) to 9 (most vigorous)]. Plants with a DMACA score ≥ 4, and plants with highest CT levels and best vigor (>6) were selected for

crosses. At least 6 top performing BC_1 individuals were selected and each was backcrossed (reciprocal crosses) to at least 5 elite non-transformed Mainstay genotypes to produce the BC_2 progenies. Likewise, the 2 top performing BC_1 genotypes from each event were pairwise crossed to produce a T_2 family (Figure 2).

1.5 Quantification of soluble and insoluble CTs in white clover leaves

Soluble CTs were routinely extracted and quantified as previously described (Peel & Dixon, 2007; Roldan et al., 2020) with slight modification. Briefly, approximately 10 mg of freeze-dried, milled plant powder was added to 0.5 mL of aqueous acetone buffer (70% (v/v) acetone, 0.5% (v/v) acetic acid), vortexed for 30 sec then sonicated in a water bath for 1 h at room temperature. Following centrifugation at 2,500 x g for 10 min, the residue was re-extracted as above, and the supernatants from both extractions were pooled and extracted with chloroform. The pellet was air-dried for later measurement of insoluble CTs. The aqueous supernatant, which contains the soluble CTs, was assayed spectrophotometrically using a Tunable Versamax Microplate Reader (Molecular Devices) at 640 nm after a 5-minute reaction with DMACA reagent (0.2 % w/v DMACA in methanol-3N HCl), with epigallocatechin (EGC, Indofine Chemical Company, Hillsborough, NJ) as the standard. EGC was initially dissolved in small amount of methanol then added with water to a final stock concentration of 3 mg/mL. Similar protocol was used to extract soluble CTs for protein binding using crude CT extract however, tissues were bulked, and extraction buffers were scaled up to produce extracts sufficient for the assay. Crude CTs were quantified, freeze dried and kept in -20°C freezer until used.

Insoluble CTs (in the pellet from soluble CT extraction mentioned above) were quantified as previously described (Pang et al., 2008). Briefly, 0.5 mL of butanol-HCl (95:5, v/v) reagent was added to the air-dried residue and the mixture was sonicated for 1h at room temperature. After centrifugation at 2,500 x g for 10 min the absorbance of the supernatant was read at 550 nm (in a microplate reader as described above). The extract was then heated at 100°C for 1 h, cooled to room temperature and the absorbance was again measured at 550 nm. The difference between the second and the first absorbance readings was converted to insoluble CT equivalents using a standard curve of purified leaf CTs from white clover (purification method described below).

1.6 Preparation of leaf samples for CT composition analysis

For NMR, the 3 primary white clover transgenic events (CTB-T_0; CTF-T_0 and CTG-T_0) were vegetatively cloned (to produce 5 clones per event) by propagating stolon tip cuttings in potting mix and grown under glasshouse conditions as described above. Green leaves (minus petioles) of different developmental stages were harvested, bulked, freeze dried, milled to a fine powder, and stored at -20°C until a total of >20 g per event had been collected for extraction. For UPLC-DAD-MS/MS, approximately 20 mg of milled, freeze-dried samples aliquoted from samples collected for CT quantitation (12 WAP) were used.

1.7 Extraction and purification of CTs from T_0 transgenic plants and their progeny

Dried leaf powder from 5 clones were pooled (~20 g each event) and was placed in a 500 mL Erlenmeyer flask equipped with a magnetic stirrer and 160 mL of 7:3 (v/v) acetone/water was added. The mixture was stirred rapidly for 30 min and then filtered using a Buchner funnel equipped with Reeve Angel filter paper (grade 202). The resultant solids were returned to the Erlenmeyer flask and extracted twice more with 160 mL of 7:3 (v/v) acetone/water. The combined filtrates were concentrated on a rotary evaporator (<40 °C), the aqueous layer diluted with CH_2Cl_2 (150 mL), and the biphasic mixture allowed to stir gently overnight. The aqueous and CH_2Cl_2 layers were separated using a separatory funnel and the aqueous layer was gently stirred with a fresh volume of CH_2Cl_2 (150 mL) for 4 h. The aqueous and CH_2Cl_2 layers were separated, and the aqueous layer was subjected to rotary evaporation (>40 °C) to remove any remaining traces of CH_2Cl_2. The aqueous layer was frozen in liquid nitrogen and freeze-dried to afford ~6 g of a tan powder. This powder was dissolved in 1:1 (v/v) methanol/water (120 mL) and Sephadex LH-20 (GE Healthcare, Marlborough, MA) was added in small portions, while stirring with a spatula, until the mixture reached the consistency of wet sand. The total amount of Sephadex LH-20 added was 33 g. This mixture was transferred to a 300 mL coarse sintered-glass Buchner filter funnel equipped with a filter paper. A mixture of methanol/water (1:1, 160 mL) was added to the resin, the mixture was stirred with a spatula, allowed to stand for 5 min and then vacuum filtered. The resin was washed in this manner 4 additional times with methanol/water (1:1, 160 mL). A mixture of acetone/water (7:3, 160 mL) was then added to the resin, the mixture was stirred with a spatula, allowed to stand for 5 min and then vacuum filtered. The resin was washed in this manner 3 additional times with acetone/water (7:3, v/v, 160 mL). The four washings were combined, concentrated on a rotary evaporator (>40 °C) to remove

the acetone, frozen in liquid nitrogen and freeze-dried to afford 372 mg of a fluffy tan solid for CTB-T_0, 285 mg for CTF-T_0 & 203 mg for CTG-T_0.

1.8 Extraction and quantification of white clover protein

Crude protein from non-transgenic white clover leaves ('Grasslands Mainstay', genotype HS227/3 R2) was obtained following published procedures (Sullivan & Zeller, 2013; Zeller et al., 2015). Fully opened trifoliate leaves (petioles excluded) were harvested and powdered in liquid nitrogen using a mortar and pestle. Powdered tissues were homogenized in 50 mM 2-[N-morpholino]ethane sulfonic acid (MES) pH 6.5 (with NaOH) extraction buffer (3 $mL.g^{-1}$ tissue). The slurry was filtered through 2 layers of muslin cloth and the filtrate was centrifuged at 4°C for 10 min at 15000 x g. The supernatant was then transferred to a 1.5 mL fresh tube and the crude extract was desalted by gel filtration chromatography using PD10 desalting columns containing Sephadex G-25 following the manufacturer's protocol (GE Healthcare Life Sciences Limited, Buckinghamshire, UK). Protein concentration was determined using the Qubit® Fluorometer (Life Technologies, Singapore) according to the manufacturer's instructions, and the protein solution stored at -80°C until required.

The bovine serum albumin (BSA) protein stock was prepared by dissolving the (BSA) powder in 50 mM MES, pH 6.5, to a concentration of 10 mg/ml. The purified or crude CT stock solution, 10 mg/mL, was prepared by dissolving lyophilized CTs in 50 mM MES buffer, pH 6.5, and then diluting them to desired working concentration with the 50 mM MES buffer.

2. Supplementary Figures and Tables

2.1 Supplementary figure

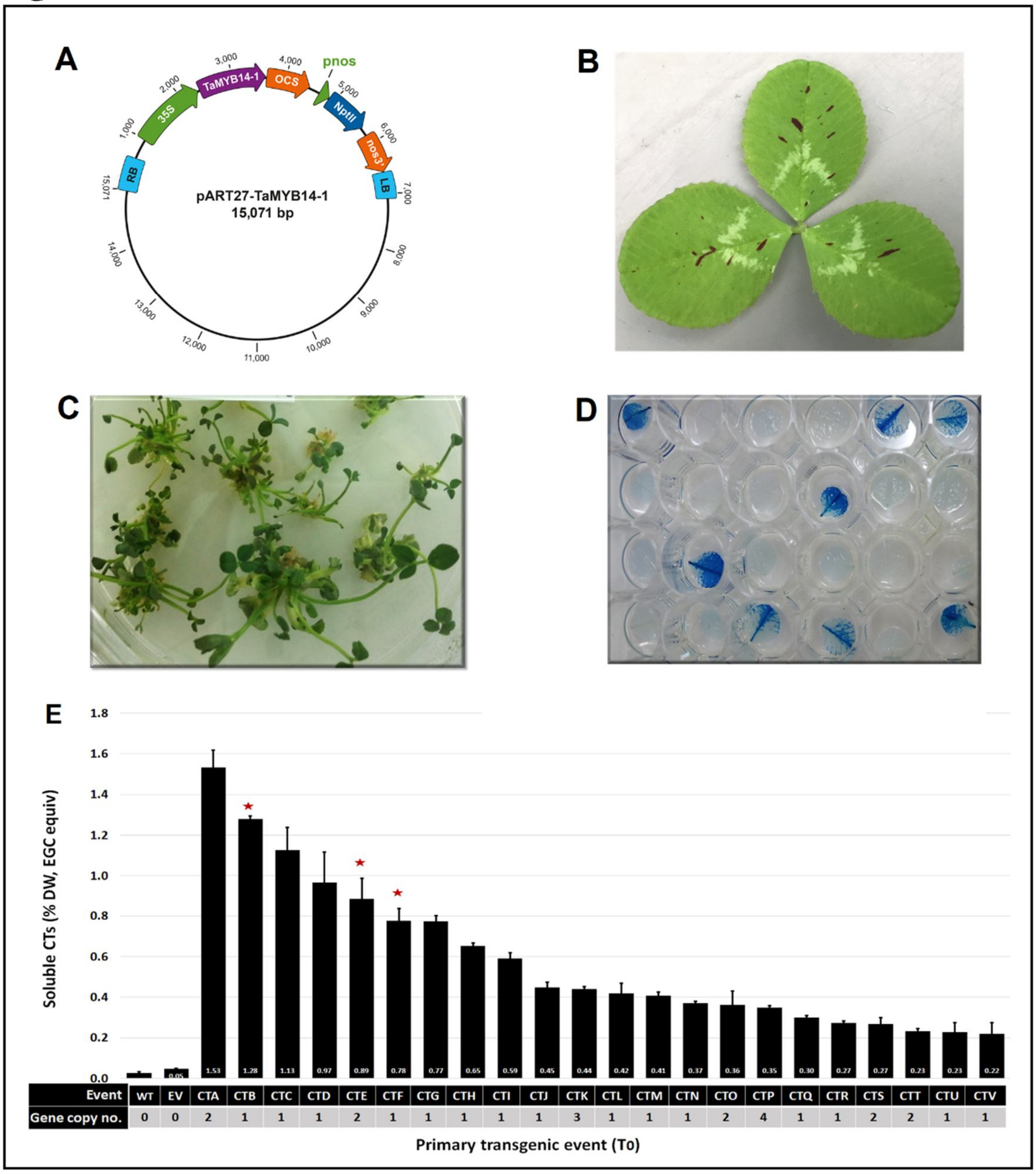

Supplementary Figure 1. Development and initial evaluation of white clover primary transgenic lines. **A)** Binary vector pART 27 containing the *TaMYB14-1* gene, **B)** trifoliate leaf of white clover 'Grasslands Mainstay' showing the 'red fleck' markings on the adaxial surface, **C)** representative image of regenerating plantlets on selection media containing 80 mg/L kanamycin, **D)** DMACA-stained leaflets showing CT negative (colorless) and CT positive (blue) leaflets, and **E)** soluble CTs in selected primary transgenic clover lines (CTA-CTV). Results from a wild type plant, plus a plant transformed with the empty vector (EV) were included as negative controls (both are cv. Mainastay). The copy number of the *TaMYB14-1* gene insertions determined using Southern hybridization and droplet digital PCR techniques are provided at the bottom of each bar. The red star indicates the primary transgenic events chosen for further breeding after initial screening for soluble CTs and segregation of T_1 progenies. The histogram and numbers at the base of each bar show the mean soluble CTs in extracts from three different plant developmental stages (4, 8 and 12 weeks after potting, WAP). Error bars represent error mean square.

Category	Score	Description of Staining Pattern
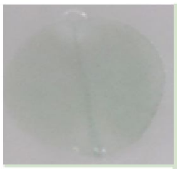	0	No staining visible by eye or Staining limited to trichomes
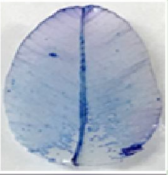	1	Staining in trichomes, leaf margin, weak spots on leaf blades and some vasculature
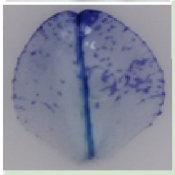	2	Punctate staining in 10-20% of leaf surface and midvein
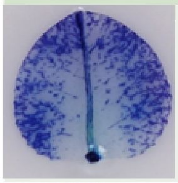	3	Punctate staining in 20-30% of leaf surface and midvein
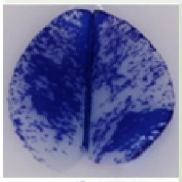	4	Punctate staining in 30-50 % of leaf surface, dark staining on midvein
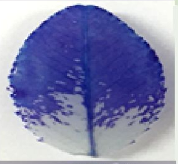	5	Staining in ~50-75 % of leaf surface, dark staining on midvein
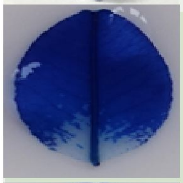	6	Dark blue staining in ~ 75-90 % of leaf surface and midvein
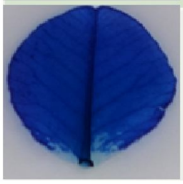	7	Dark blue staining in >90% of leaf surface, areas not stained limited to leaf base

Supplementary Figure 2 Criteria used for scoring DMACA-stained white clover leaflets for condensed tannins.

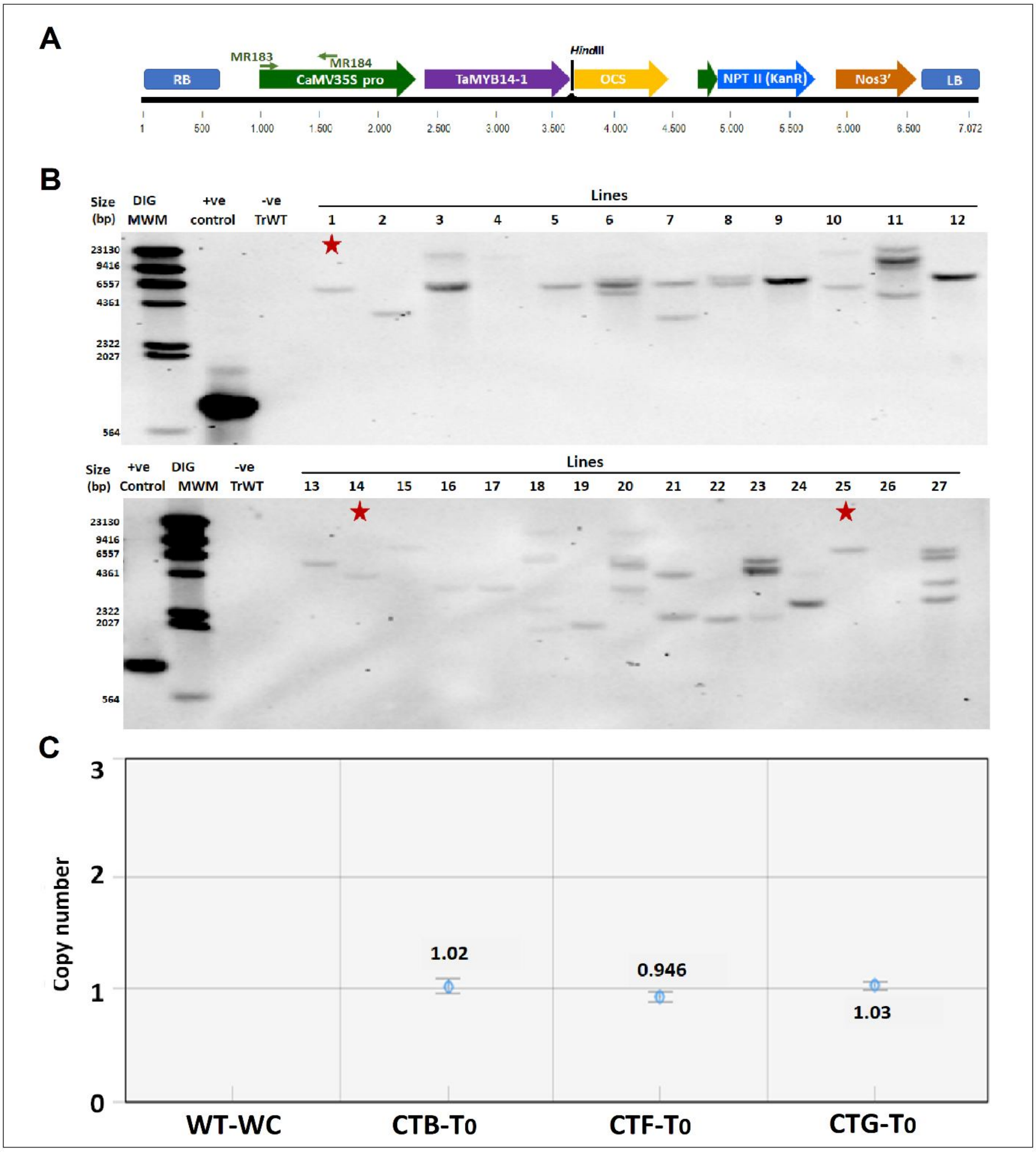

Supplementary Figure 3. **A)** Diagram of the T-DNA showing the position of primers used to confirm T-DNA integration by PCR and to synthesize the Southern hybridisation probe, **B)** Southern blot hybridisation used to check the transgene copy number in the primary transformation events. White clover genomic DNA (10 µg) was digested with *Hin*dIII. The red asterisks show the CTB, CTF and CTG events in lanes 1, 14 and 25, respectively. The marker is a DIG-labelled DNA Molecular Weight Marker II (Roche, Cat # 11218590910), the positive and negative controls are pART27-TaMYB14-1 plasmid DNA and genomic DNA isolated from non-transformed Mainstay white clover, respectively; both digested with *Hind*III, and **C)** result of copy number variation (CNV) analysis of the three selected primary transgenic events by digital droplet PCR.

Supplementary Figure 4. PCR screening of selected white clover genotypes for vector contamination beyond the T-DNA borders. **A)** Map of the pART27-TaMYB14-1 binary vector showing the locations of primers designed to identify vector fragments in transgenic plants by PCR, and **B)** results of PCR analysis of control plasmid DNA (i) plus genomic DNA from 3 primary transgenic events (ii-iv). Lane numbers in (i) correspond with hypothetical PCR amplicons in A.

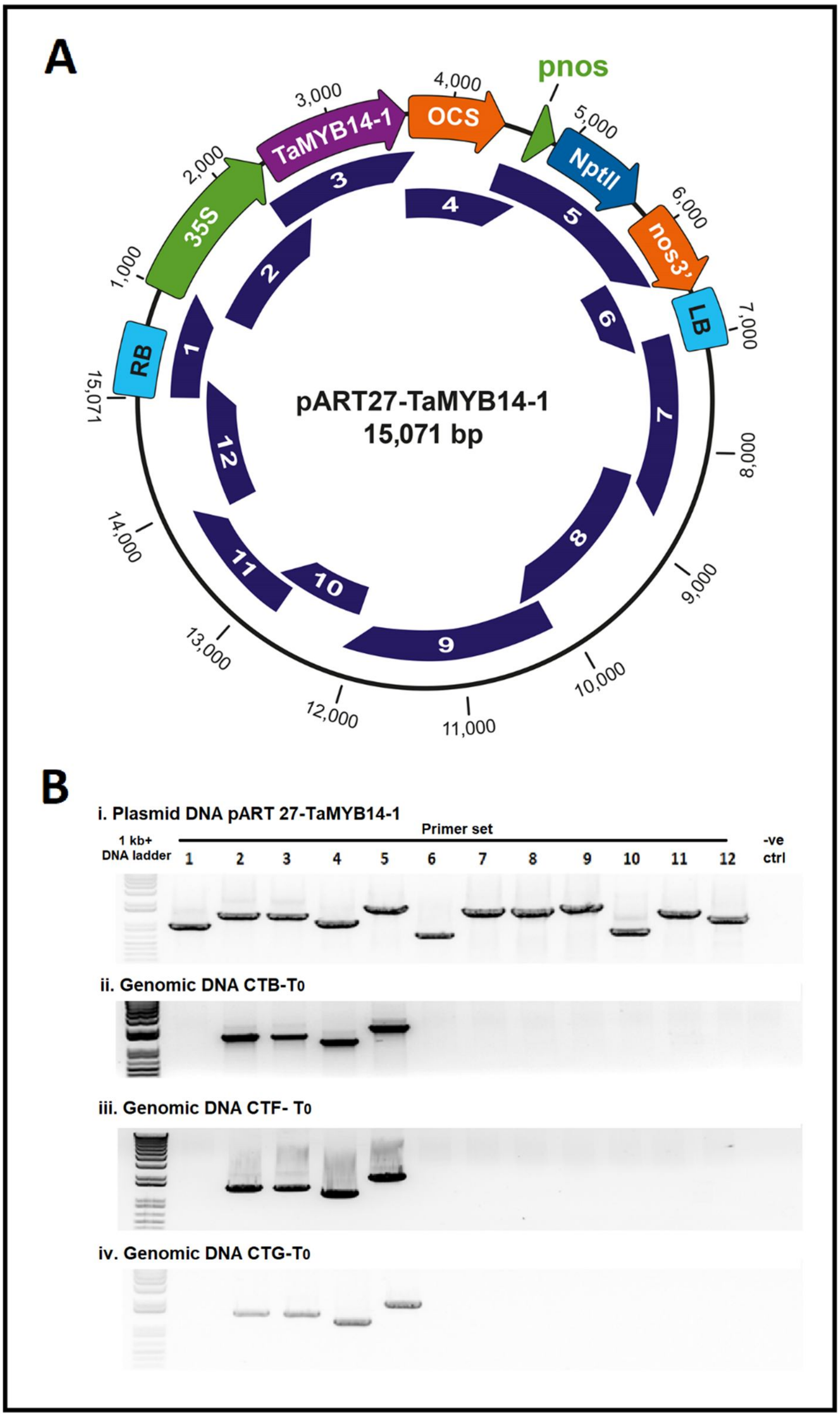

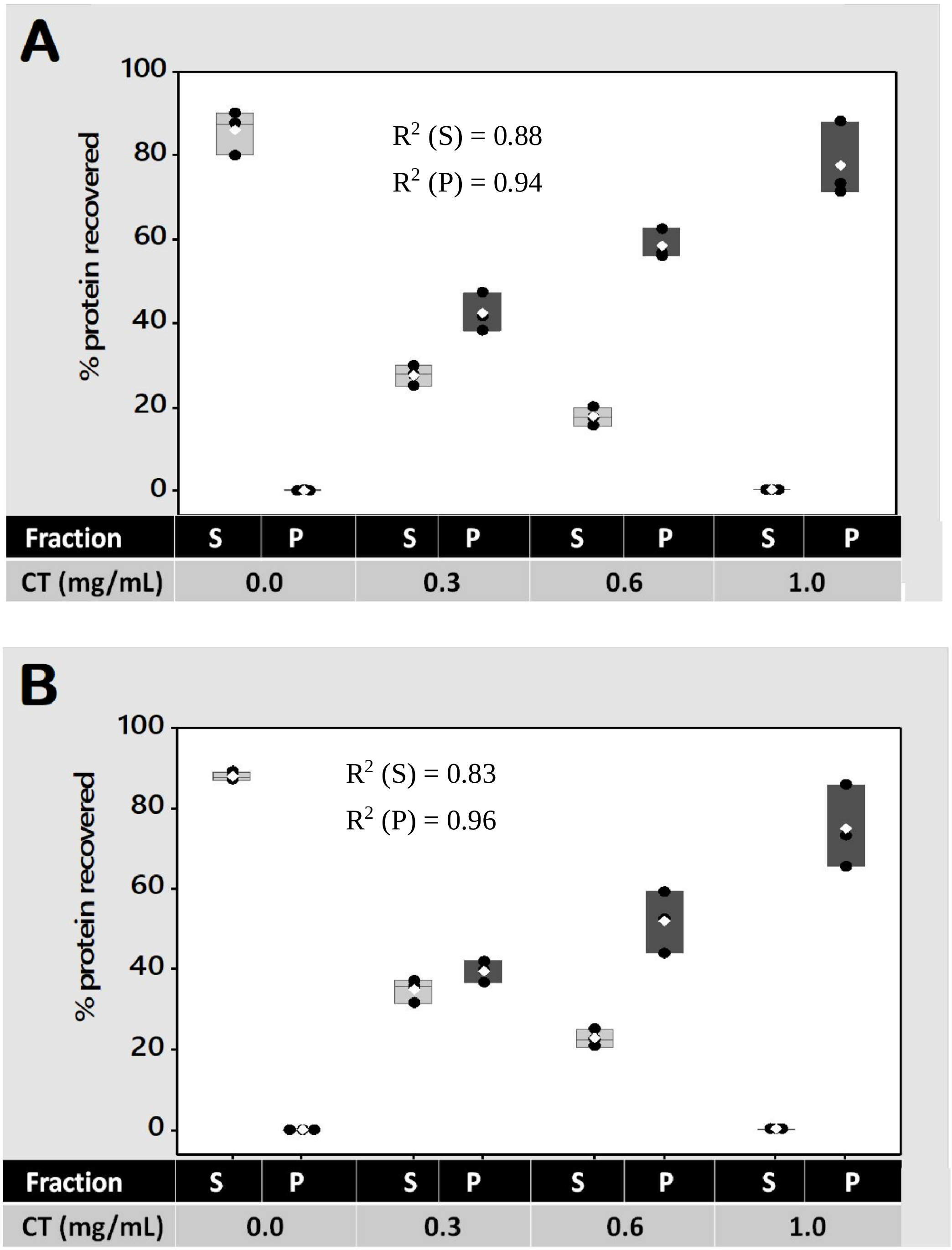

Supplementary Figure 5. Percent of **A)** BSA and **B)** white clover protein (1 mg/mL protein) recovered after incubation for 20 minutes with different concentrations of white clover CTs *in vitro* in 50 mM of 2-(N-morpholino) ethane sulfonic acid (MES) at pH 6.5. CTs from the leaves of three primary transgenic events (CTB -T_0, CTF-T_0 and CTG- T_0) were purified as described in methods section (1.7 Extraction and purification of CTs from T_0 transgenic plants and their progeny

) and were used as biological replicates. Each data point in A and B represents a biological replicate; S = supernatant, P= pellet.

2.2 Supplementary Tables

Supplementary Table 1A. Summary of white clover transformation data and selection of primary transgenic events (T_0) for breeding

Description	Number
Cotyledons inoculated	25,000
Plants that survived Kanamycin (80 mg/L) selection	162
CT positive events (determined by PCR and DMACA staining)	57
Events with strong CT expression (determined by DMACA)	22
Events with a single T-DNA insertion (determined by Southern hybridisation & Droplet digital PCR)	15
Events selected for test crossing to determine T-DNA segregation ratios in progeny	8
Primary transgenic lines (events) selected for further breeding	3

Supplementary Table 1B. Segregation of T_1 progeny from testcrosses between 8 primary transgenic events and elite non-transformed 'Grasslands Mainstay' genotypes. The segregation ratio of the T-DNA was determined in leaflets from 4 WAP seedlings stained with DMACA. A score >1 or <1 was deemed to be T-DNA positive or negative respectively.

Event	Number of crosses	Total plants analyzed	CT positive plants	CT negative plants	% DMACA positive	χ^2 value	P-value
**CTA-T_0**	2	20	8	12	39.58	0.80	0.371
**CTB-T_0[1]**	28	372	194	178	52.08	0.68	0.407
**CTC-T_0**	39	475	9	466	1.92	439.68	<.001
**CTD-T_0**	20	240	18	222	7.50	173.40	<.001
**CTE-T_0**	5	72	46	26	63.33	5.56	0.018
**CTF-T_0[1]**	20	228	124	104	54.52	2.12	0.156
**CTG-T_0[1]**	24	288	138	150	47.92	0.50	0.480
**CTH-T_0**	19	240	65	175	21.19	50.42	<.001

[1] - Selected for further breeding

Supplementary Table 2. Oligonucleotide sequences used in DNA sequencing, PCR and probe synthesis

Name	Orientation	Short description	Purpose	Sequence (5'-3')
MR183	Forward	CaMV35S	PCR, probe synthesis for Southern hybridisation & DNA sequencing	CCCACAAAAATCTGAGCTTA
MR184	Reverse	CaMV35S	PCR, probe synthesis for Southern hybridisation & DNA sequencing	TGCATCTTTAACCTTCTTGG
MR194	Forward	LB pART27	PCR / Vector contamination check	CCGAATTATCAGCCTTCTTA
MR195	Reverse	LB pART27	PCR / Vector contamination check	GAATAAGGGACAGTGAAGAA
MR200	Forward	Fragment 1 / RB	PCR & DNA sequencing	CGTTCGGATATTTTCGTGGAG
MR201	Reverse	Fragment 1/ CaMV35S promoter	PCR & DNA sequencing	CTCAGATTTTTGTGGGATTGGA
MR202	Forward	Fragment 2 /CaMV35S promoter	PCR & DNA sequencing	AGCTCTCCCATATCGACCTG
MR203	Reverse	Fragment 2 /CaMV35S promoter	PCR & DNA sequencing	TGTCTTGTATTCCAACCTAAGG
MR204	Forward	Fragment 3 / TaMYB14-1 gene	PCR & DNA sequencing	GACCCTTCCTCTATATAAGGAA
MR205	Reverse	Fragment 3 / TaMYB14-1 gene	PCR & DNA sequencing	TCTCATTAAAGCAGGACTCTAG
MR206	Forward	Fragment 4 / OCS terminator	PCR & DNA sequencing	GAGGAAGTACTAGGAGAATGAA
MR207	Reverse	Fragment 4 / OCS terminator	PCR & DNA sequencing	GCTTAGCTCATTAAACTCCAG
MR208	Forward	Fragment 5/ NOS promoter	PCR & DNA sequencing	TTACCCAACTTAATCGCCTTG
MR209	Reverse	Fragment 5/ NOS promoter	PCR & DNA sequencing	CGGCAACAGGATTCAATCTTAA
MR210	Forward	Fragment 6/ NOS terminator	PCR & DNA sequencing	GTTTTCAATAAGGACGAGATGG
MR211	Reverse	Fragment 6/ left border	PCR & DNA sequencing	TTCTAAAGAAATAGCGCCAC
MR212	Forward	Fragment 7 / vector	PCR / Vector contamination check	CTGTCGATCTTGAGAACTATG
MR213	Reverse	Fragment 7 / Vector	PCR / Vector contamination check	GTGTCCAGATAATCCACCTTAT
MR214	Forward	Fragment 8 / vector	PCR / Vector contamination check	CTCCATCAAGAAGAGCGACT
MR215	Reverse	Fragment 8 / vector	PCR / Vector contamination check	TCCTGTTTGAAGACCGACAG
MR216	Forward	Fragment 9 / vector	PCR / Vector contamination check	GAAGAAAGCTCCAGAGATGTT
MR217	Reverse	Fragment 9 / vector	PCR / Vector contamination check	GATGAACGAAATAGACAGATCG
MR218	Forward	Fragment 10 / vector	PCR / Vector contamination check	ATGAGTAAACTTGGTCTGACAG
MR219	Reverse	Fragment 10 / vector	PCR / Vector contamination check	GAGCTTCATCCACTAAAACA
MR231	Forward	Fragment 11 / vector	PCR / Vector contamination check	CAAAATGTGGCTTTACTCTCG
MR224	Reverse	Fragment 11 / vector	PCR / Vector contamination check	CTTATGGAGTTGTCGTAGTTG
MR220	Forward	Fragment 12 / vector	PCR / Vector contamination check	GTCTTCCCTATGACTACTCC
MR221	Reverse	Fragment 12 / vector	PCR / Vector contamination check	CTGGATCTCGCCTTCAATCC

Supplementary Table 3. Composition of condensed tannins in substrates used in rumen fluid *in vitro* studies to evaluate protein degradation and fermentation products

Variables	$CTG\text{-}T_2$ (3755)	$CTG\text{-}T_2$ (3764)	WT WC inflorescences	WT WC Leaves
Soluble CTs (% DW)[1]	2.38	1.61	1.87	0.01
Total CTs (soluble + insoluble, % DW)[2]	5.64	4.95	4.91	0.06
% Procyanidin (PC)[3]	15.75	14.91	1.8	n.d
% Prodelphinidin (PD)[3]	84.25	85.09	98.2	n.d
Mean degree of polymerization (mDP)[3]	11	10	15	n.d

[1] Extraction using 70% (v/v) acetone: 0.5% (v/v) acetic acid (Peel & Dixon, 2007)

[2] Butanol:HCl (95:5, v/v) method used for insoluble CTs (Pang & Dixon 2008)

[3]Analysed using using Waters Xevo UPLC-DAD-MS/MS system (Salminen, 2018)

n.d = not detected

Supplementary Table 4. Nutrient composition of the substrates used in protein degradation and methane emission analysis. The samples were subjected to Near Infrared Reflectance Spectroscopy (NIRS) by Analytical Research Laboratories (ARL, Ravensdown, Napier, NZ)**.**

Variables[1]	WT WC leaves	CTG-T_2 (3755)	CTG-T_2 (3764)	WT WC inflorescences
ADF (% w/w DM)	15.8	18.4	15.5	18.1
Ash (% w/w DM)	11.7	13.5	13.5	14.6
CP (% w/w DM) **	29.6	26.5	28.4	25.5
Lipid (% w/w DM)	3.9	3.1	3.1	4.2
ME (est) (MJ/kg DM)	12.7	11.9	12	11.8
NDF (% w/w DM)	31.2	28.4	27.8	32.3
OMD (*in vivo*; % w/w DM)	> 85	81.2	81.9	80.8
SSS (% w/w DM)	8.2	7.1	7.4	8.7

[1] - ADF, Acid detergent fibre; CP, crude protein; NE, metabolizable energy; NDF, neutral detergent fibre; OMD, organic matter digestibility; SSS, soluble sugars and starch; DM, dry matter

Note: Mention of trade names or commercial products in this article is solely for the purpose of providing specific information and does not imply recommendation or endorsement by the U.S. Department of Agriculture. USDA is an equal opportunity provider and employer.

References

Bio-Rad. (2017). *QX200 Droplet Reader and QuantaSoft Software Instruction Manual.*

Chen, B. C. M., & McManus, M. T. (2006). Expression of 1-aminocyclopropane-1-carboxylate (ACC) oxidase genes during the development of vegetative tissues in white clover (Trifolium repens L.) is regulated by ontological cues [Article]. *Plant Mol Biol, 60*(3), 451-467. https://doi.org/10.1007/s11103-005-4813-3

Doyle, J., & Doyle, J. (1987). A rapid DNA isolation procedure for small quantities of fresh leaf tissue. *Phytochemical Bulletin, 19*(1), 11-15.

Li, Y. G., Tanner, G., & Larkin, P. (1996). The DMACA-HCl protocol and the threshold proanthocyanidin content for bloat safety in forage legumes [Article]. *Journal of the Science of Food and Agriculture, 70*(1), 89-101. https://www.scopus.com/inward/record.uri?eid=2-s2.0-0003015963&partnerID=40&md5=31cd1461dadbbf4d8904a26307decbeb

Mazaika, E., & Homsy, J. (2014). Digital Droplet PCR: CNV Analysis and Other Applications [Article]. *Current protocols in human genetics, 82*, 7.24.21-27.24.13. https://doi.org/10.1002/0471142905.hg0724s82

Pang, Y., Peel, G. J., Sharma, S. B., Tang, Y., & Dixon, R. A. (2008). A transcript profiling approach reveals an epicatechin-specific glucosyltransferase expressed in the seed coat of Medicago truncatula. *Proceedings of the National Academy of Sciences of the United States of America, 105*(37), 14210-14215. https://doi.org/10.1073/pnas.0805954105

Peel, G. J., & Dixon, R. A. (2007, May 2007). Detection and quantification of engineered proanthocyanidins in transgenic plants. *Natural Products Communications, 2*(10), 1009 - 1014. http://digital.library.unt.edu/ark:/67531/metadc488160

Roldan, M., Islam, A., Dinh, P. T. Y., Leung, S., & McManus, M. T. (2016). Phosphate availability regulates ethylene biosynthesis gene expression and protein accumulation in white clover (Trifolium repens L.) roots [Article]. *Bioscience Reports, 36*(6), Article e00411. https://doi.org/10.1042/bsr20160148

Roldan, M. B., Cousins, G., Fraser, K., Hancock, K. R., Collette, V., Demmer, J., Woodfield, D. R., Caradus, J. R., Jones, C., & Voisey, C. R. (2020). Elevation of Condensed Tannins in the Leaves of Ta-MYB14-1 White Clover (Trifolium repens L.) Outcrossed with High Anthocyanin Lines [Article]. *Journal of Agricultural and Food Chemistry, 68*(10), 2927-2939. https://doi.org/10.1021/acs.jafc.9b01185

Salminen, J. P. (2018). Two-Dimensional Tannin Fingerprints by Liquid Chromatography Tandem Mass Spectrometry Offer a New Dimension to Plant Tannin Analyses and Help to Visualize the Tannin Diversity in Plants [Article]. *Journal of Agricultural and Food Chemistry, 66*(35). https://doi.org/10.1021/acs.jafc.8b02115

Sullivan, M. L., & Zeller, W. E. (2013). Efficacy of various naturally occurring caffeic acid derivatives in preventing post-harvest protein losses in forages [Article]. *Journal of the Science of Food and Agriculture, 93*(2), 219-226. https://doi.org/10.1002/jsfa.5781

Voisey, C. R., White, D. W. R., Dudas, B., Appleby, R. D., Ealing, P. M., & Scott, A. G. (1994). Agrobacterium-mediated transformation of white clover using direct shoot organogenesis. *Plant Cell Reports, 13*(6), 309-314. https://doi.org/10.1007/bf00232627

Zeller, W. E., Sullivan, M. L., Mueller-Harvey, I., Grabber, J. H., Ramsay, A., Drake, C., & Brown, R. H. (2015). Protein precipitation behavior of condensed tannins from Lotus pedunculatus and Trifolium repens with different mean degrees of polymerization [Article]. *Journal of Agricultural and Food Chemistry, 63*(4), 1160-1168. https://doi.org/10.1021/jf504715p
